# Discovering the potential active ingredients of Qi-Yu-San-Long decoction for anti-oxidation, inhibition of non-small cell lung cancer based on the spectrum-effect relationship combined with chemometric methods

**DOI:** 10.3389/fphar.2022.989139

**Published:** 2022-10-19

**Authors:** Mengwen Huang, Ruijuan Li, Mo Yang, An Zhou, Hong Wu, Zegeng Li, Huan Wu

**Affiliations:** ^1^ Key Laboratory of Xin’an Medicine, Ministry of Education, Anhui University of Chinese Medicine, Hefei, China; ^2^ Anhui Province Key Laboratory of Chinese Medicinal Formula & Anhui Province Key Laboratory of Research and Development of Chinese Medicine, Hefei, China; ^3^ Key Laboratory of Traditional Chinese Medicine for Prevention and Treatment of Major Pulmonary Diseases, Department of Education of Anhui Province, Hefei, China

**Keywords:** Qi-Yu -San-Long decoction, UPLC-Q/TOF-MS fingerprints, antioxidant, anti-NSCLC, spectrum-effect relationship

## Abstract

Qi-Yu-San-Long decoction (QYSLD), a traditional Chinese medicine (TCM) prescription, consisting of ten types of herbal medicine which has significant clinical efficacy in the treatment of non-small cell lung cancer (NSCLC). However, the bioactive ingredients of QYSLD remain unclear, due to their “multi-ingredients” and “multi-targets” features. This study aimed to construct a spectrum-effect correlation analysis model and screen the potential active components of QYSLD. A fingerprint method based on ultra-high performance liquid chromatography-quadrupole time-of-flight mass spectrometry (UPLC-Q/TOF-MS) was developed and validated to obtain seventy common peaks of ten batches of QYSLD. The results of methodological evaluation, including precision, repeatability and stability, were less than 8.19%. In terms of linearity, eleven common components did not reach the linear standard (R^2^ < 0.99), they were removed before spectrum-effect relationship analysis. After treated with ten batches of QYSLD, the results of DPPH and FRAP assays ranged from 1.59 to 5.50 mg mL^−1^ and 143.83–873.83 μmol L^−1^, respectively. Meanwhile, the cell viabilities of A549 cells treated with QYSLD samples ranged from 21.73% to 85.71%. The relative healing rates ranged from 21.50% to 44.46%. The number of migrated and invaded cells ranged from 12.00 to 68.67 and 7.67 to 27.00, respectively. Then, the potential active components of QYSLD were screened through spectrum-effect relationship constructed by grey correlation analysis (GRA), partial least squares regression (PLSR) and backpropagation neural network (BP-ANN). The results were as follow: 1) eight ingredients of QYSLD were relevant to DPPH free radical scavenging ability; 2) nine ingredients were relevant to FRAP; 3) six ingredients were relevant to inhibit the proliferation ability of A549 cells; 4) twenty-two ingredients were relevant to inhibit the horizontal migration ability; 5) five ingredients were relevant to inhibit the vertical migration ability; 6) twelve ingredients were relevant to inhibit the invasion ability. Confirmatory experiments showed that compared with the unscreened ingredients, the potential active ingredients screened by the spectrum-effect relationship had better antioxidant and anti-NSCLC effects. In general, this study found the potential active ingredients in QYSLD. Meanwhile, the established method provided a valuable reference model for the potential active ingredients of TCM.

## Graphical Abstract

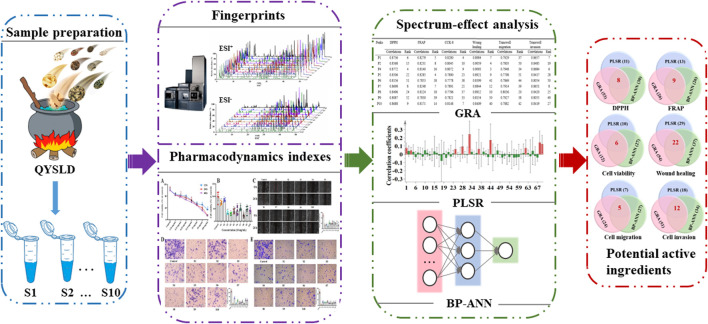

## 1 Introduction

Lung cancer is one of the most commonly diagnosed cancer (11.4% of total cases) and is the leading cause of cancer death (18.0% of the total cancer deaths) (Sung et al., 2021). Lung cancer can be divided into small cell lung cancer (SCLC) and non-small cell lung cancer (NSCLC, the most common disease type) by histopathology. It is well established that NSCLC genesis is a multi-stage process, in which normal cells, under various stresses, undergo a large number of genetic and epigenetic mutations to gradually form cancer cells and eventually develop into tumors (Liu et al., 2020). In addition, redox homeostasis is a basic requirement for performing various normal cellular functions. When reactive oxygen species (ROS) are out of balance, the potential for tumor formation is increased through the activation of oncogenic signaling pathways, immune disorders, DNA mutations, metastasis, angiogenesis, tumor microenvironment, and telomere extension (Kirtonia et al., 2020). Besides, oxidative stress, as the main driving force of malignant transformation of tumor cells, can enhance the potential metastasis of cancer cells. The increase in ROS is important for tumor cells to sustain a highly metastatic phenotype (Piskounova et al., 2015).

Traditional Chinese medicine (TCM) provides an unrivalled resource for discovering promising drug candidates and treating various diseases. However, the bioactive ingredients of Chinese herbal medicine commonly remain unclear, due to their “multi-ingredients” and “multi-targets” features. Different combinations of chemical components of TCM play different pharmacodynamic roles. It is of great significance for revealing the pharmacodynamic substances of TCM to establish a correlation analysis model through multi-dimensional indexes and screen the active components. Qi-Yu-San-Long decoction (QYSLD) is made up of ten Chinese medicines of *Astragalus mongholicus* Bunge (Huangqi, HQ), *Solanum nigrum* L. (Longkui, LK), *Hedyotis diffusa* Willd. (Baihuasheshecao, BHSSC), *Coix lacryma-jobi* L. (Yiyiren, YYR), Curcuma *phaeocaulis* Valeton (Ezhu, EZ), *Polygonatum odoratum* (Mill.) Druce (Yuzhu, YZ), *Scolopendra subspinipes mutilans* L.Koch (Tianlong, TL), *Pheretima aspergillum* (E. Perrier) (Dilong, DL), *Euphorbia helioscopia* L. (Zeqi, ZQ) and *Fritillaria cirrhosa* D.Don (Chuanbeimu, CBM), combined in the ratio of 15:10:10:10:5:5:3:3:3:3 by weight. The above botanical and animal drugs belonged to Fabaceae, Solanaceae, Rubiaceae, Poaceae, Zingiberaceae, Asparagaceae, Scolopendridae, Megascolecidae, Euphorbiaceae and Liliaceae, respectively. It is a clinical prescription mainly used in the treatment of NSCLC (Tong et al., 2018a). The research group previously explored the mechanism by which QYSLD inhibits NSCLC from the perspectives of genes, proteins and endogenous small molecule metabolites (Tong et al., 2016; Wu et al., 2018a; Tong et al., 2018b). And the overall chemical composition of QYSLD has also been studied (Huang et al., 2021), but the active ingredients have not been fully elucidated.

Spectrum-effect relationship is one of the popular methods to study the active ingredients of TCM (Li et al., 2019; Wu et al., 2021). It can effectively combine the fingerprint spectrum with the results of pharmacodynamics research through data processing and analysis methods, which solves the deficiencies of focusing on the ingredients and ignoring the pharmacological efficacy of TCM in the past (Zheng et al., 2021; Zhang et al., 2022). Up to now, the analytical techniques, including liquid chromatography-ultraviolet detector (LC-UV) (Li W. et al., 2020), liquid chromatography-evaporative light scattering detector (LC-ELSD) (Zhang et al., 2021), infrared spectroscopy (Li H. et al., 2020), capillary electrophoresis (Ma et al., 2017), have been used to develop fingerprint of TCM. Among them, LC-UV was one of the most commonly used method which has the advantages of cost-effective, simple and easy to implement. However, LC-UV could not meet the analysis requirements when the samples contained alkaloids, saponins and other compounds with weak UV absorption. Although LC coupled with other detectors, such as ELSD, could partly solve this problem, but the sensitivity of ELSD might not be sufficient to detect trace compounds with strong activity. Therefore, a more comprehensive and sensitive method is needed to construct fingerprints of TCM.

With the rapid development of high-resolution mass spectrometry (HRMS), quadrupole-time-of-flight (Q-TOF), orbitrap, linear-ion-trap and ion-trap-TOF have been widely used in the analysis of TCM components due to their enhanced capacity to measure the exact mass of analytes and its fragmentations (Ji et al., 2018; Fu et al., 2019; Chen et al., 2021). Compared with LC-UV and LC-ELSD, LC-HRMS has the advantages as follows: 1) unaffected by compounds without UV absorbing groups; 2) highly sensitivity and resolution; 3) structural characterization while separating components. Particularly, UPLC-Q/TOF-MS provides a full-information tandem mass spectrometry mode (MS^E^), which can achieve low collision energy and high collision energy alternate scanning to obtain highly accurate information of parent ion and daughter ion information (Wu et al., 2018b; Wu et al., 2020). In recent years, it has been widely used in chemical composition analysis (Xu et al., 2020; Huang et al., 2021), metabolism study (Fu et al., 2021; Li et al., 2022) and metabolomics research (Zhan et al., 2020; Xu et al., 2021; Wu et al., 2022). For fingerprints, the data information provided by the spectra at low energy of UPLC-Q/TOF-MS can be used for the establishment of fingerprints and the precise fragment ion information provided by the spectra at high energy can be used to identify the structural information of components.

However, UPLC-Q/TOF-MS is rarely used in the construction of TCM fingerprint directly, mainly because of the exported data, including the retention time and peak area data, cannot be directly used for analysis by the currently developed similarity evaluation software. In order to solve this problem, the UPLC-Q/TOF-MS fingerprint data was exported into a self-built TXT file. The processing of UPLC-Q/TOF-MS fingerprint data was mainly divided into two parts in the self-built TXT file. The first part was the processing of chromatogram data, that is, the retention time and peak intensity of components were rearranged according to the peak order of components to form dataset 1. The second part was the processing of fingerprint integral data, that is, the peak height and the integrated peak area were rearranged according to the retention time to form dataset 2. Then dataset one and two were merged, and the encoding of the TXT file was set to ANSI, which was successfully used for fingerprint similarity evaluation. In this research, the established UPLC-Q/TOF-MS fingerprint method was used to obtain common peaks of ten batches of QYSLD, and then the potential active ingredients with the effects of antioxidant and anti-NSCLC in QYSLD were screened by the spectrum-effect relationship. This study revealed that different combinations of chemical components of TCM have different effects, and provided reference for the discovery of active ingredients of TCM.

## 2 Materials and methods

### 2.1 Reagents and materials

The human lung adenocarcinoma cell line A549 was provided by the cell bank in the Biological Sciences Institute of Shanghai. Fetal bovine serum (FBS) was obtained from Biological Industries (Kibbutz Beit Haemek, Israel). Dulbecco’s modified Eagle’s medium (DMEM) was purchased from Procell (Wuhan, China). Cell counting kit-8 (CCK-8) was supplied from BioSharp (Hefei, China). Matrigel were purchased from Corning (Corning, NY, United States). LC-grade methanol, acetonitrile and formic acid were obtained by TEDIA (Cincinnati, Ohio, United States), MERCK & Co., Inc. (Darmstadt, Germany) and Aladdin (Shanghai, China) respectively. 2,2-diphenyl-1-picrylhydrazyl (DPPH) and 2,4,6-tripyridyl-s-triazine (TPTZ) were purchased from Macklin (Shanghai, China). Ultrapure water was produced with a Milli-Q water purification system (Millipore, Billerica, MA, United States). The reference standards, including solamargine, calycosin-7-*O*-*β*-D-glucoside, monotropein, deacetyl asperulosidic acid, astragaloside II and curcumol were obtained from Chengdu MUST Bio-Technology Co., Ltd. (Chengdu, China). Chlorogenic acid, notoginsenoside R1 and peimisine were obtained from Shanghai yuanye Bio-Technology Co., Ltd (Shanghai, China). Ten types of herbal medicine in QYSLD (HQ, LK, BHSSC, YYR, EZ, YZ, TL, DL, ZQ, and CBM) were purchased from Tongrentang Chinese Medicine (Beijing, China) and belonged to Faaceae, Solanaceae, Rubiaceae, Poaceae, Zingiberaceae, Asparagaceae, Scolopendridae, Megascolecidae, Euphorbiaceae and Liliaceae, respectively. All herbs were identified by associate professor Qingshan Yang, an expert in pharmacognosy at Anhui University of Traditional Chinese Medicine and deposited at the Herbarium of the Anhui University of Chinese Medicine, Hefei, China (Herbarium code: ACM, voucher numbers: 20049, 20051, 20077, 20040, 20083, 20056, 20091, 20105, 20032, 20075). The details of drug materials are provided in Supplementary Table S1.

### 2.2 Sample preparation

Ten batches of QYSLD extracts were prepared using conventional extraction method and orthogonal experimental design. HQ (30 g), LK (20 g), BHSSC (20 g), YYR (20 g), EZ (10 g), YZ (10 g), TL (6 g), DL (6 g), ZQ (6 g) and CBM (6 g) were soaked in 1.34 L distilled water (1/10, w/v) for 1 h, boiled for 1.5 h and then filtered; the drug residue was boiled for 40 min in 1.07 L distilled water (1/8, w/v). The two extracts were combined, concentrated, freeze-dried to powder and marked as S10. According to the above traditional decoction method, the solid-liquid ratio, soaking time, boiling time and decoction times were selected as influencing factors and each factor had three levels to design an orthogonal table. Then, nine batches of QYSLD samples were boiled according to the conditions in Supplementary Table S2 and labeled S1-S9.

For UPLC-Q/TOF-MS analysis, each freeze-dried powder was accurately weighed and dissolved in 90% methanol at a concentration of 100 mg mL^−1^ (equivalent to the concentration of the raw medicinal materials), then ultrasonicated for 0.5 h, filtered with 0.22 μm microporous filter membrane.

### 2.3 UPLC-Q/TOF-MS conditions

For UPLC analysis, chromatographic separation was performed using an ACQUITY I-Class UPLC system (Waters Corporation, Milford, Mass, United States) combined with a ZORBAX RRHD Eclipse Plus C18 column (1.8 μm, 2.1 × 100 mm). The injection volume was 2 μL. The temperature of the column was kept at 35°C and the flow rate was 0.2 ml min^−1^. Mobile phase solvent A (water with 0.1% formic acid, V/V) and solvent B (acetonitrile) were used. The gradient profile was performed as follows: 0–13 min, 97%–87% A; 13–17 min, 87%–85% A; 17–27 min, 85%–75% A; 27–32 min, 75%–65% A; 32–42 min, 65%–40% A; 42–47 min, 40%–3% A; 47–48 min, 3%–97% A; 48–52 min, 97% A.

For Q/TOF-MS conditions, the detector was carried out on Waters Xevo G2-XS Q/TOF mass spectrometer (Waters Corporation, Milford, Mass, United States) with electrospray ionization (ESI). The MS conditions were as follows: the capillary voltage was 3.0 kV/–2.5 kV; desolvation gas (N_2_) flow rate was 600 L h^−1^; desolvation temperature was 400°C; source temperature was 120°C; cone gas flow was 40 L h^−1^; the mass range was 50–1200 Da. Leucine-enkephalin was chosen as the lock mass compound for the ESI^+^ mode [(M + H)^+^ = 556.2771] and ESI^−^ mode [(M−H)^–^ = 554.2615] to ensure accurate MS analysis. Data were acquired through MS^E^ (lower-energy collision voltage was 6 V and the higher-energy scan ranging from 20 to 35 V). All data were collected by Masslynx 4.1 software (Waters Corporation, Milford, Mass, United States).

### 2.4 Establishment and evaluation of UPLC-Q/TOF-MS fingerprints of Qi-Yu-San-Long decoction

In order to ensure that the methodology of the established UPLC-Q/TOF-MS fingerprint was reliable, quality control (QC) samples were selected for methodological verification. QC samples (400 mg mL^−1^) were prepared by pooling the same amount of QYSLD of ten samples together. Then, linearity was verified by diluting QC samples serially (2, 4, 8, 16, 32, 64 times) with 90% methanol. Notoginsenoside R1 was selected as internal standard (IS). The ratio of the peak area (each analyte to IS) were plotted versus the concentrations of QC sample for calibration curves of each common peak and the assessment was based on the obtained coefficients of determination (*R* ^*2*^). Seventy peaks were chosen for calculate the relative standard deviations (RSDs) of peak area and retention time. The repeatability was evaluated by RSDs of the peak area and retention time, which was calculated from six samples in parallel of QC samples (100 mg mL^−1^); stability was evaluated by RSDs of the peak area and retention time at different time points (0, 4, 8, 12, 16, and 24 h) from QC samples; precision was assessed by RSDs of the peak area and retention time for six consecutive injections with QC samples.

Ten batches of QYSLD samples were determined under the conditions of ‘2.3’. Retention time and peak area of common peaks were exported. The IS peak was selected as the reference peak to calculate the relative retention time (RRT) and relative peak area (RPA) of common peaks. Then, coefficients of variance (CVs%) of RRT and RPA of each component in ten batches of QYSLD were calculated. Similarity evaluation was performed by the Similarity Evaluation System for Chromatographic Fingerprints of TCM (Version 2004A; Beijing, China).

### 2.5 Evaluation of antioxidant activity of Qi-Yu-San-Long decoction

#### 2.5.1 1,1-Diphenyl-2-picrylhydrazyl radical assay

The DPPH assay was determined referring to a method reported by Li (Li et al., 2012). QYSLD samples with different concentrations (0.3125, 0.625, 1.25, 2.5, 5, 10, and 20 mg mL^−1^) were prepared and mixed with equal volume of DPPH (0.05 mg mL^−1^). After being placed and reacted under dark for 40 min at 37°C. The optical density (OD) values were measured at a wavelength of 517 nm using an auto-microplate reader (Molecular Devices, Sunnyvale, California, United States). IC50 values were calculated using GraphPad Prism 8.0 software. Ascorbic acid was used as positive control. The scavenging rate was calculated by the following formula:

$$DPPH\;scavenging\;rate\left(\%\right)=\left[1-\left({OD}_{sample}-{OD}_{blank}\right)/{OD}_{control}\right]\times 100\%$$

OD_sample_ represents the OD value which measured after sample solution mixed with DPPH solution; OD_blank_ represents the OD value which measured after sample solution mixed with 90% methanol; OD_control_ represents the OD value which measured after DPPH solution mixed with 90% methanol.

#### 2.5.2 FRAP assay

The FRAP experiment was evaluated according to a method described by Benzie IF (Benzie and Strain, 1999). Then, 0.3 ml of each batch of QYSLD samples (20 mg mL^−1^) was mixed with 2.7 ml FRAP working solution preheated to 37°C (1 part of 20 mmol L^−1^ FeCl_3_‧6H_2_O; 10 part of 300 mmol L^−1^ sodium acetate with pH 3.6; one part of 10 mmol L^−1^ TPTZ) and reacted for 10 min. The absorbance was measured at 593 nm. Blank groups (90% methanol) and positive groups (ascorbic acid) were performed in parallel. Ferrous sulfate solutions at concentrations of 25, 50, 100, 200, 400, and 800 μmol L^−1^ were used to establish the standard calibration curve. The antioxidant activity was expressed as the concentration of Fe^2+^.

### 2.6 Evaluation of anti-non-small cell lung cancer activity of Qi-Yu-San-Long decoction

#### 2.6.1 CCK-8 experiment

QYSLD sample solutions under item ‘2.2’ were blown dry with nitrogen and reconstituted with DMEM for cell assays. A549 cells were routinely cultured in DMEM with 10% FBS in 37°C incubator with 5% CO_2_. A549 cells were seeded in a 96-well plate at a density of 5×10^3^ cells/100 µL/well. A549 cells were treated with QYSLD (S10) at different concentrations (0, 1.56, 3.13, 6.25, 12.5, 25, 50,and 100 mg mL^−1^) for different hours (12, 24, and 48 h). Next, the culture medium was discarded, and the cells in each well were incubated with 100 µL DMEM containing 10% CCK-8 reagent for 1 h and measured by auto-microplate reader (450 nm). IC50 value was calculated using GraphPad Prism 8.0 software. Then, the appropriate concentration and time were selected as the culture conditions, and the above operations were repeated to determine the survival rate of A549 cells which were treated with each batch of QYSLD samples.

#### 2.6.2 Wound healing test

Cell suspension containing 2×10^5^/ml A549 cells was seeded into a 6-well plate. The monolayer of cells was scratched with the tip of a pipette when cells grew to approximately 85% and washed carefully twice with phosphate buffer saline (PBS) to remove cell debris. Then, A549 cells were cultured with DMEM containing QYSLD sample solutions (S1-S10) without FBS as the administration group and DMEM without FBS as the control group. Cells were incubated at 37°C with 5% CO_2_ for 24 h. The condition of scratches was observed using the CKX41 inverted microscope (Olympus Corporation, Tokyo, Japan) at 0 h and 24 h (100×). ImageJ software (National Institutes of Health) was used to measure the healing area and calculate the relative healing rate.

#### 2.6.3 Transwell assay

The effects of QYSLD (S1-S10) on the vertical migration and invasion of A549 cells were assessed in transwell chambers containing polycarbonate membranes with 8 μm pore size. The upper chambers of migration and invasion are different, namely with or without Matrigel. A549 cells were added to each upper chamber of 24-well plate at a concentration of 4×10^4^ cells/mL. Meanwhile, 500 μL 15% FBS-DMEM medium and medicated medium were added to lower chambers, which were incubated for 24 h. Then, cells that penetrated the polycarbonate membrane were fixed with 4% formaldehyde, stained with crystal violet, and subsequently photographed using a microscope (200×).

### 2.7 Spectrum-effect relationship

The peak areas of common components with linear *R* ^*2*^ greater than 0.99 of ten batches of QYSLD were used as ‘spectrum’, and the antioxidant (DPPH and FRAP assays) and anti-NSCLC activities (A549 cells proliferation, horizontal migration, vertical migration and invasion abilities) were used as ‘effects’. Spectrum-effect relationship was established *via* grey correlation analysis (GRA), partial least squares regression (PLSR) and backpropagation neural network (BP-ANN) combined with mean impact value (MIV) algorithm to explore the active components in QYSLD.

#### 2.7.1 Grey correlation analysis

GRA was performed by MTALAB software (2016a MathWorks Inc., Natick, Mass, United States) with ‘effects’ as reference sequences and ‘spectrum’ as the compared sequences. The spectrum-effect relationship was explained by calculating the gray value correlation degree. The higher the correlation degree between chemical components in QYSLD and ‘effects’, the higher the grey correlation degree value. In this study, chemical components with gray value over 0.8 were considered to be closely related to ‘effects’. The formula was described as follows:

$$\xi_{i}\left(k\right)=\frac{\min_{s}\underset{t}{\;\min}\left|x_{0}\left(t\right)-x_{s}\left(t\right)\right|+\rho \cdot \underset{s}{\max\;}\max_{t}\left|x_{0}\left(t\right)-x_{s}\left(t\right)\right|}{\left|x_{0}\left(k\right)-x_{i}\left(k\right)\right|+\rho \cdot \underset{s}{\max\;}\max_{t}\left|x_{0}\left(t\right)-x_{s}\left(t\right)\right|}$$

$$r_{i}=\frac{1}{N}\Sigma \xi_{i}\left(k\right)$$

Where $\xi_{i}\left(k\right)$ is the correlation coefficient between the comparison sequence ( $x_{i}$ ) and the reference sequence ( $x_{0}$ ) on the k-th index; ρ represents the resolution coefficient, which is 0.5; $\underset{s}{\min\;}\min_{t}\left|x_{0}\left(t\right)-x_{s}\left(t\right)\right|$ and $\max_{s}\underset{t}{\;\max}\left|x_{0}\left(t\right)-x_{s}\left(t\right)\right|$ are the minimum difference and maximum difference of the two stages, respectively; $r_{i}$ represents the grey correlation degree between the comparison sequence ( $x_{i}$ ) and the reference sequence ( $x_{0}$ ); N is the number of data in the comparison sequence.

#### 2.7.2 Partial least squares regression

PLSR is a statistical method that projects the independent variable (‘spectrum’) and dependent variable (‘effects’) into a new space to find linear regression model. Independent variables with statistical significance were screened by setting the variable influence on projection (VIP)>1 as the condition, combined with the correlation coefficient to explain the degree of influence between the independent variable and the dependent variable. This model was implemented using SIMCA-P 14.1 (Umetrics, Malmo, Sweden).

#### 2.7.3 Backpropagation neural network combined with mean impact value algorithm

BP-ANN is an artificial neural network of error back propagation algorithm with feedforward network architecture that includes input layer, hidden layer and output layer. As one of the best indicators to evaluate the influence of variables on the results in neural network application, through the combination of neural network and mean impact value (MIV), the independent variables that have a greater influence on the ‘effects’ are screened, so as to achieve the variable screening of neural network (Zhang et al., 2018). Independent variables were added or subtracted by 10% respectively on the basis of original data to form two simulation samples, and the difference of the simulation results is the impact value (IV) of changing independent variables on the output results. The mean difference value of IV is the MIV. The symbol of MIV represents the correlation direction, and the magnitude of absolute value represents the degree of correlation. Calculation of MIV was done using MTALAB software. The model parameter (*R* ^*2*^) is a statistical indicator reflecting the correlation between the actual value and the predicted value.

### 2.8 Evaluation of potential active components

#### 2.8.1 Evaluation of antioxidant components

In the DPPH evaluation experiment, calycosin-7-*O*-*β*-D-glucoside and deacetyl asperulosidic acid, which are closely related to DPPH scavenging capability, were selected for evaluation according to the results of spectrum-effect relationship. Meanwhile, two other components (peimisine and astragaloside II) with weak correlations with this ‘effect’ were selected for parallel experiments. The above components were diluted with 90% methanol to various concentration gradients (12.5, 25, 50, 100, 200, and 400 μg mL^−1^), and the specific experimental procedures were performed with reference to ‘2.5.1’.

In the FRAP evaluation experiment, deacetyl asperulosidic acid and monotropein, which are closely related to FRAP, were selected for evaluation according to the results of spectrum-effect relationship. Meanwhile, two other components (peimisine and astragaloside II) with weak correlations with this ‘effect’ were selected for parallel experiments. The above components were individually dissolved with 90% methanol at a concentration of 100 μg mL^−1^, and the specific experimental procedures were performed with reference to ‘2.5.2’.

#### 2.8.2 Evaluation of potential active components for inhibiting cell viability

The effect of potential active components on the proliferation of A549 cells was assessed by CCK-8 assay. Calycosin-7-*O*-*β*-D-glucoside, curcumol and solamargine were selected among the potential active components based on the results of the spectrum-effect relationship for evaluation. Meanwhile, chlorogenic acid with poor correlations with the ‘effects’ was selected for parallel experiments. The specific experimental steps were performed with reference to ‘2.6.1’.

### 2.9 Statistical analysis

All experimental data were expressed as the mean ± SD and analyzed with the statistical software GraphPad Prism 8.0. One-way analysis of variance (ANOVA) followed by Student-Newman-Keuls post hoc test were used for Multiple groups statistical analysis. *p* < 0.05 was considered statistically significant.

## 3 Results

### 3.1 UPLC-Q/TOF-MS fingerprints of Qi-Yu-San-Long decoction

#### 3.1.1 Method evaluation

As shown in Supplementary Table S3, the linear regression of P3, P11, P27, P31, P33, P40, P41, P45, P53, P57, and P69 was poor (*R* ^*2*^ < 0.99), while the other common peaks showed good linear regression (*R* ^*2*^ > 0.99). The RSDs of RRT and RPA of seventy peaks for precision were less than 0.91% and 7.72%, respectively. RSD values of RRT and RPA for repeatability were lower than 0.88% and 7.87%, respectively. As for stability test, RSDs of RRT and RPA were below 1.02% and 8.19%, respectively. It indicated that the instrument has good precision and reproducibility, suitable for fingerprint analysis. Method evaluation results for UPLC-Q/TOF-MS fingerprints of QYSLD were detailed in Supplementary Table S3.

#### 3.1.2 Similarity analysis

In the positive ion mode, the similarities between the S1-S10 spectra and the reference fingerprint ranged from 0.882 to 0.974, among which the similarity of S1 was 0.882, and the other batches were more than 0.940. In the negative ion mode, the similarities between the S1-S10 spectra and the reference fingerprint were 0.797–0.967, among which the similarity of S1 was 0.797, and the remaining batches were more than 0.810. The detailed similarity evaluation results were shown in Supplementary Table S4.

#### 3.1.3 UPLC-Q/TOF-MS fingerprints

**FIGURE 1:**
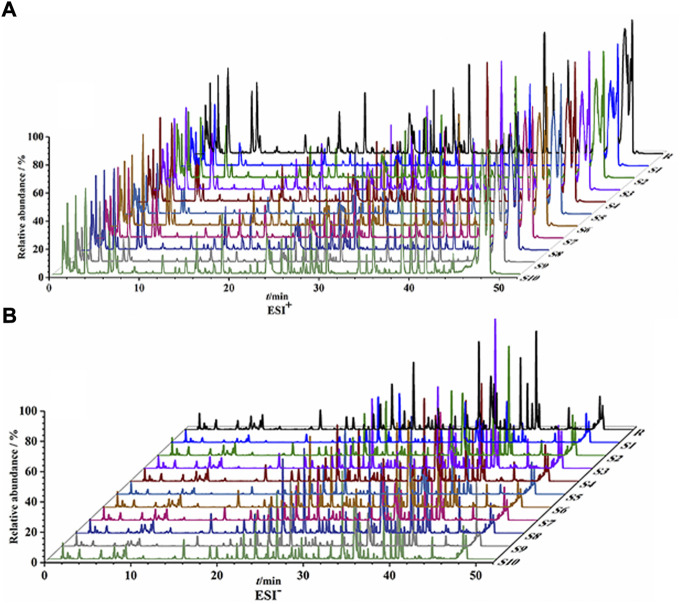
UPLC-Q/TOF-MS fingerprints of ten batches of QYSLD. **(A)** positive ion mode; **(B)** negative ion mode.

**FIGURE 2:**
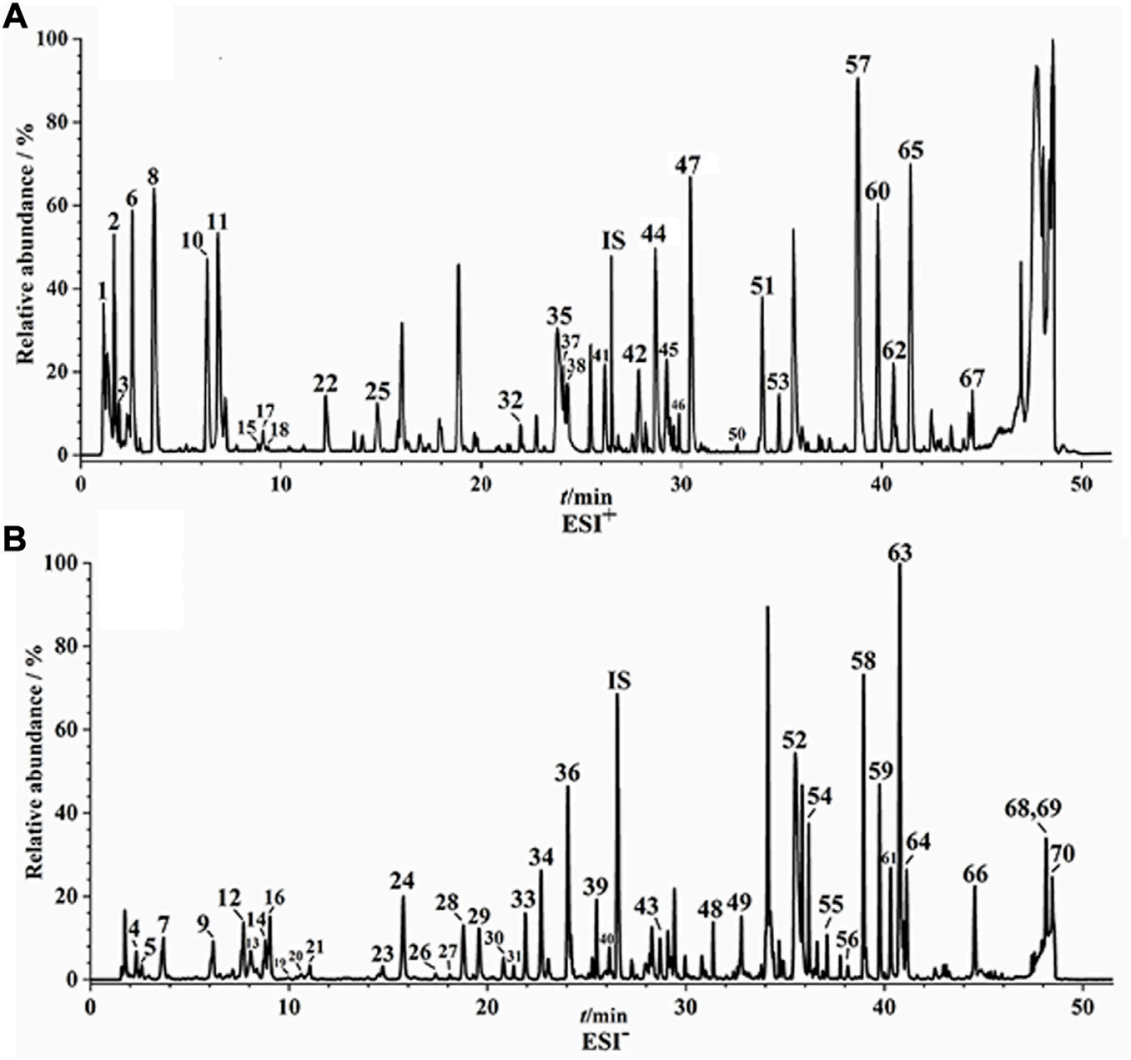
The reference fingerprints of QYSLD. **(A)** positive ion mode; **(B)** negative ion mode.

UPLC-Q/TOF-MS fingerprints (Figure 1) and reference fingerprints (Figure 2) were obtained after similarity evaluation of the chemical profiles of ten batches of QYSLD. According to the retention time of the reference spectra, peaks of each batch of QYSLD were compared, and seventy peaks were finally selected as common components. Among them, the IS peak was selected as the reference peak to calculate RRT and RPA of each common peak. The results included in Supplementary Table S5,S6 showed that the CVs% of RRT were 0.03%–1.98% and 0.00%–1.78% in the positive ion and negative ion modes, respectively. Meanwhile, the CVs% of RPA were 17.06%–153.29%, 9.37%–86.06%, respectively.

On the basis of previous research, seventy common peaks were characterized (Huang et al., 2021). The detailed information of seventy components of QYSLD, such as retention time, chemical formulas and names, precise precursor and product ions and component names, were shown in Supplementary Table S7.

### 3.2 Antioxidant effect of Qi-Yu-San-Long decoction

DPPH and FRAP experiments were used to evaluate the antioxidant capacity of ten batches of QYSLD. In the DPPH experiment, the IC50 values of ten batches of QYSLD were in the range of 1.59–5.50 mg mL^−1^. Besides, in the FRAP experiment, the FRAP values were in the range of 143.83–873.83 μmol L^−1^. The results showed that S3 had strong antioxidant effect, while S2 showed weak antioxidant effect. Meanwhile, the antioxidant levels of the remaining batches of QYSLD were ranked differently in the DPPH and FRAP experiments. The IC50 and FRAP values are presented in Table 1.

**TABLE 1:** Antioxidant activity of ten batches of QYSLD (mean ± SD, *n* = 3).

Sample	DPPH IC50	FRAP the concentration of Fe^2+^
S1	1.71 ± 0.12 (mg·mL^−1^)	463.83 ± 6.64 (μmol·L^−1^)
S2	5.50 ± 0.20 (mg·mL^−1^)	143.83 ± 1.56 (μmol·L^−1^)
S3	1.59 ± 0.09 (mg·mL^−1^)	873.83 ± 2.12 (μmol·L^−1^)
S4	3.19 ± 0.11 (mg·mL^−1^)	327.58 ± 8.31 (μmol·L^−1^)
S5	3.24 ± 0.18 (mg·mL^−1^)	251.33 ± 5.62 (μmol·L^−1^)
S6	2.65 ± 0.08 (mg·mL^−1^)	372.58 ± 4.82 (μmol·L^−1^)
S7	3.41 ± 0.03 (mg·mL^−1^)	390.92 ± 3.68 (μmol·L^−1^)
S8	2.43 ± 0.67 (mg·mL^−1^)	382.33 ± 4.64 (μmol·L^−1^)
S9	3.51 ± 0.89 (mg·mL^−1^)	208.42 ± 4.68 (μmol·L^−1^)
S10	3.40 ± 0.07 (mg·mL^−1^)	352.17 ± 3.54 (μmol·L^−1^)
Positive control	3.29 ± 0.12 (mg·mL^−1^)	503.83 ± 0.59 (μmol·L^−1^)

### 3.3 Anti-non-small cell lung cancer activity of Qi-Yu-San-Long decoction

#### 3.3.1 Qi-Yu-San-Long decoction inhibited A549 cells proliferation

**FIGURE 3:**
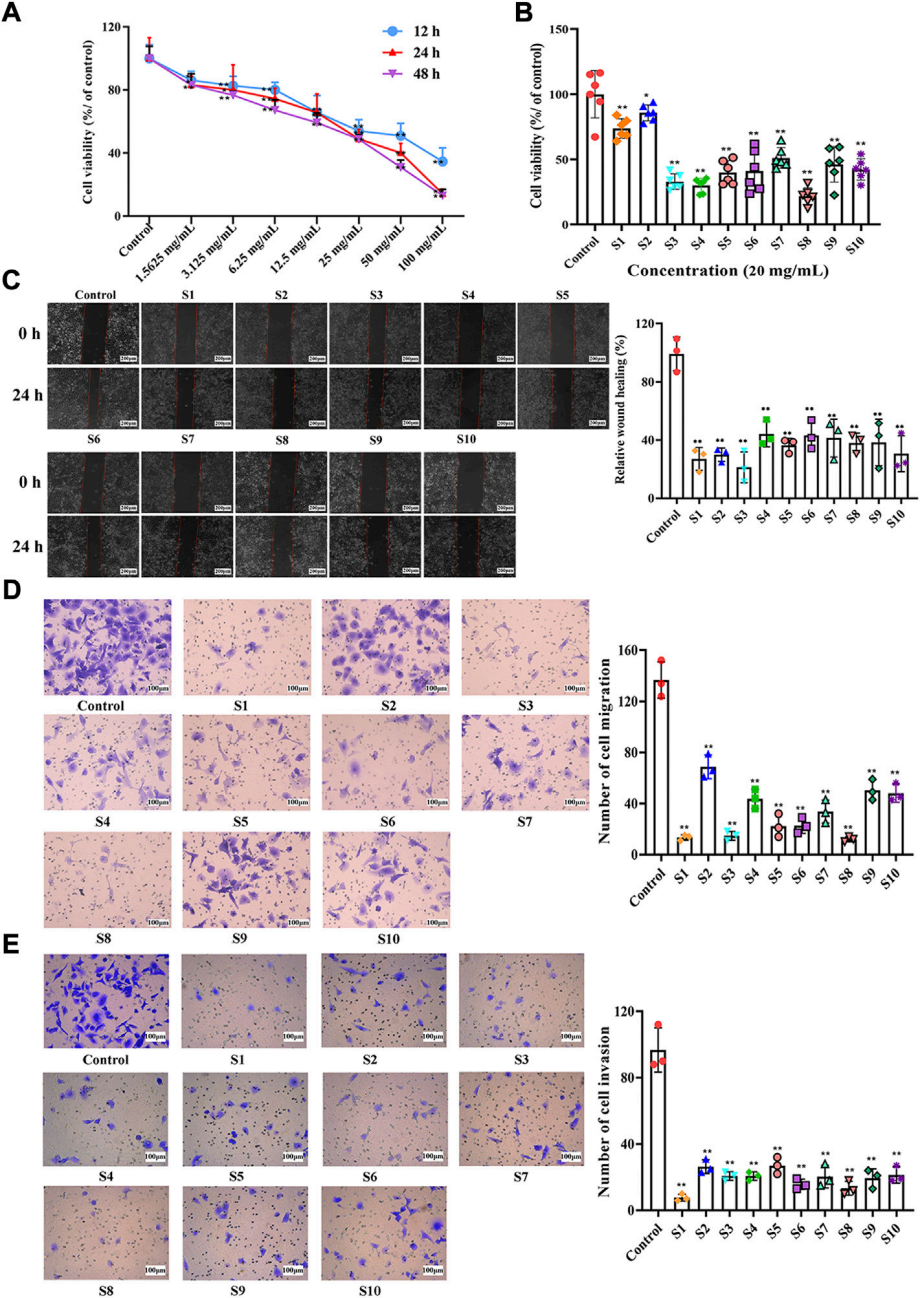
Effects of QYSLD samples on the proliferation, horizontal migration and vertical migration and invasion of A549 cells. **(A)** Effect of S10 on the proliferation of A549 cells at different time points and concentrations was determined by CCK-8 kit (*n* = 6). **(B)** Effects of ten batches of QYSLD on the proliferation activity of A549 cells were determined by CCK-8 kit (*n* = 6). **(C)** Effects of ten batches of QYSLD on the horizontal migration ability of A549 cells were determined by wound healing test (*n* = 3, the scale bar is 200 μm). **(D)** Effects of ten batches of QYSLD on the vertical migration activity of A549 cells were determined by transwell migration assay (*n* = 3, the scale bar is 100 μm). **(E)** Effects of ten batches of QYSLD on the invasion activity of A549 cells were determined by transwell invasion assay (*n* = 3, the scale bar is 100 μm). [**(C–E)**: The left side represents the cell graph under the microscope, the right side represents the histogram after quantification]. Data are represented as mean ± SD. *p < 0.05, **p < 0.01 versus control group.

The proliferation of A549 cells was evaluated by CCK-8 assay. A549 cells were treated with different concentrations (0, 1.56, 3.13, 6.25, 12.5, 25, 50, and 100 mg mL^−1^) of QYSLD (S10) for 12, 24, and 48 h. As shown in Figure 3A, the inhibitory action of QYSLD on A549 cells was dose-dependent, and the IC50 values of 12, 24, and 48 h were 40.45, 22.56, and 17.13 mg mL^−1^, respectively. In order to reduce the error in the preparation, 20 mg mL^−1^, which is close to 22.56 mg mL^−1^, as the concentration and 24 h as the treatment time to determine the cell viability of A549 cells treated with ten batches of QYSLD samples. As shown in Figure 3B, the survival rates ranged from 21.73% to 85.71%, among which S8 had the strongest inhibitory ability and S2 was the weakest.

#### 3.3.2 Qi-Yu-San-Long decoction inhibited the horizontal migration of A549 cells

The wound healing assay assessed the horizontal migration ability of A549 cells (Figure 3C). At 0 h, the wound areas of all groups were roughly the same. After 24 h of culture, the wound healing rate of each batch of QYSLD was significantly lower than that in control group (*p* < 0.01) with a range of 21.50%–44.46%. S3 had the slowest healing rate in the administration groups, indicating that S3 had the strongest ability to inhibit the horizontal migration of A549 cells, followed by S1.

#### 3.3.3 Qi-Yu-San-Long decoction inhibited the vertical migration and invasion of A549 cells

Transwell *in vitro* migration and invasion assays were used to evaluate the vertical migration (Figure 3D) and invasion abilities (Figure 3E) of A549 cells, respectively. Compared with the control group, the number of cells in the QYSLD administration groups (S1-S10) was significantly reduced (*p* < 0.01), suggesting that ten batches of QYSLD administration groups could significantly inhibit the vertical migration and invasion abilities of A549 cells (*p* < 0.01). The number of migrated and invaded cells ranged from 12.00 to 68.67 and 7.67 to 27.00, respectively. Further, S1 and S8 showed stronger inhibitory effects than the other batches.

### 3.4 Spectrum-effect relationship

#### 3.4.1 Grey correlation analysis

For the antioxidant part, fifty-three peaks in the DPPH experiment showed a good degree of correlation with gray values ranging from 0.8008 to 0.8889. Twenty-six peaks in the FRAP assay had a gray value greater than 0.8 and the range was 0.8004–0.8487. For the anti-NSCLC active fraction, the gray scale level between common compounds and pharmacodynamic indexes were in the range of 0.6138–0.9339. Considering the peaks with gray values greater than 0.8, there were twelve peaks in the CCK-8 experiment, fifty-four peaks in the wound healing experiment, twenty-four peaks in the transwell migration and fifty-one peaks in the transwell invasion. Gray scale results are shown in Supplementary Table S8.

#### 3.4.2 Partial least squares regression analysis

**FIGURE 4:**
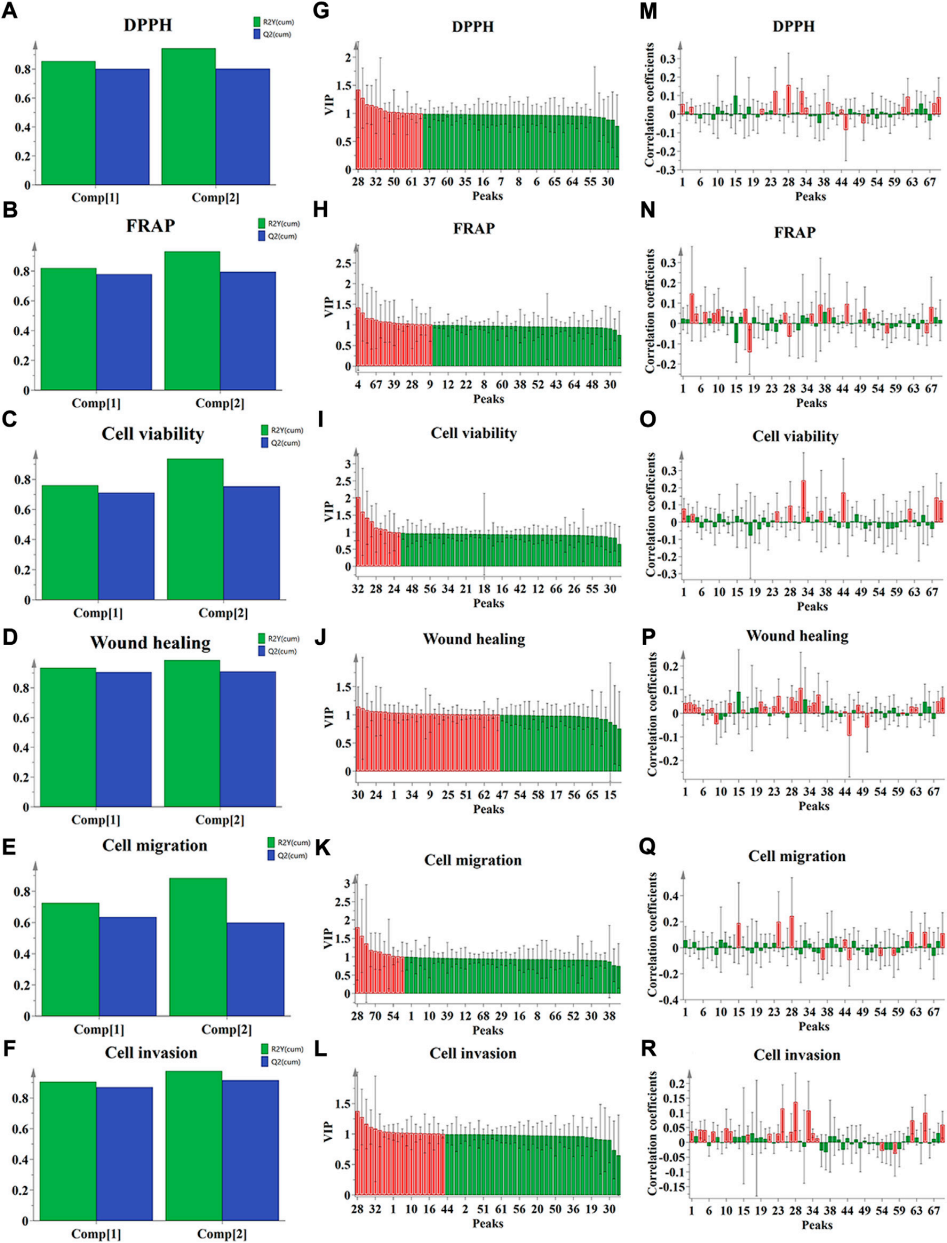
The results of spectrum-effect relationship analysis by PLSR. **(A–F)** Summary of fit plot; **(G–L)** correlation coefficients between the seventy common peaks and the antioxidant, anti-NSCLC activities, respectively, and **(M–R)** VIP values.

As shown in Figures 4A–F, the parameters of *R* ^2^ *X*, *R* ^2^ *Y*, and *Q* ^2^ in PLSR models were 0.932, 0.943, and 0.803 (for DPPH assay); 0.934, 0.932, and 0.795 (for FRAP assay); 0.935, 0.937, and 0.753 (for CCK-8 assay); 0.929, 0.987, and 0.910 (for wound healing assay); 0.930, 0.886, and 0.599 (for transwell migration assay); 0.934, 0.975, and 0.916 (for transwell invasion assay), respectively. The above parameters indicated that the established PLSR models have satisfactory prediction and explanation abilities. Then, the potential bioactive ingredients with the value of VIP> 1 were filtered out and indicated as red in fit plot of PLSR (Figure 4G–L). As shown in Figure 4M–R, the positive contribution order of seventy common peaks of QYSLD to DPPH radical scavenging was P28>, P24>, P32>, P62>, P70>, P39>, P68>, P1>, P4>, P61>, P34, and the order of FRAP was P4>, P46>, P37>, P67>, P39>, P50>, P17>, P10>, P7>, P26>, P9>, P5>, and P35. In addition, P1, P4, P24, P28, P32, P37, P44, P62, P68, and P70 were positively correlated and VIP> 1 in the CCK-8 experiment. Twenty-nine peaks showed higher correlation levels in the wound healing experiment, suggesting that the ability of cell healing may be weakened with the increase in these compounds. In the tranwell experiment, seven components were positively correlated with vertical migration ability, and eighteen components were positively correlated with invasion ability. The specific correlation coefficient and VIP values are shown in the Supplementary Table S9.

#### 3.4.3 Backpropagation neural network combined with mean impact value algorithm

**FIGURE 5:**
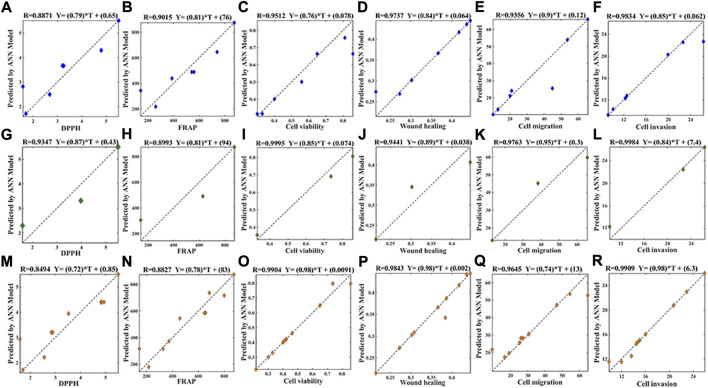
Model evaluation of BP-ANN on the training data set **(A)**: DPPH, *R* ^2^ = 0.8871; **(B)** FRAP, *R* ^2^ = 0.9015; **(C)** cell viability, *R* ^2^ = 0.9512; **(D)** wound healing, *R* ^2^ = 0.9737; **(E)** cell migration, *R* ^2^ = 0.9356; **(F)** cell invasion, *R* ^2^ = 0.9834), test data set **(G)**: DPPH, *R* ^2^ = 0.9347; **(H)** FRAP, *R* ^2^ = 0.8993; **(I)** cell viability, *R* ^2^ = 0.9995; **(J)** wound healing, *R* ^*2*^ = 0.9441; **(K)** cell migration, *R* ^*2*^ = 0.9763; **(L)**:cell invasion, *R* ^*2*^ = 0.9984) and the whole set **(M)**: DPPH, *R* ^2^ = 0.8494; **(N)**: FRAP, *R* ^2^ = 0.8827; **(O)**: cell viability, *R* ^*2*^ = 0.9904; **(P)**: Wound healing, *R* ^2^ = 0.9843; **(Q)**: cell migration, *R* ^2^ = 0.9645; **(R)**: cell invasion, *R* ^2^ = 0.9909), the nonlinear fitting performance of 70-3-1 ANN model.

As shown in Figure 5, the *R* ^2^ of values in the training data set (Figures 5A–F), the test data set (Figure 5G–L) as well as the whole data set (Figure 5M–R) were all greater than 0.84, suggesting that the BP-ANN models had good nonlinear fitting effect. Based on the above BP-ANN models, the MIVs between the seventy common peaks and the antioxidant or anti-NSCLC activities were obtained (Supplementary Table S10). The components with MIV> 0 represent positive correlation with effects. Among them, thirty components and twenty-six components were related to DPPH free radical scavenging and FRAP results, respectively; twenty-seven components, thirty-seven components, twenty-seven components, thirty-four components were associated with A549 cell proliferation, horizontal migration, vertical migration and invasion abilities, respectively.

#### 3.4.4 Shared potentially active ingredients of a single experiment were screened by combined three chemometrics

**FIGURE 6:**
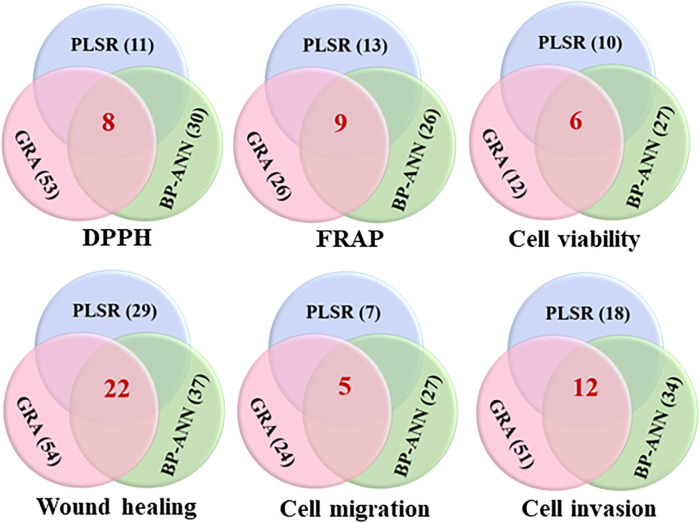
Venn diagrams of the shared potentially active ingredients in QYSLD were screened by GRA, PLSR and BP-ANN combined with MIV algorithm. (The red number represents the quantity of potential active ingredients after the intersection of three chemometrics).

To improve the reliability of forecast results of the spectrum-effect relationship, shared active ingredients of a single experiment were screened by combined three chemometrics. As shown in Figure 6, it was found that eight components (P1, P4, P24, P28, P34, P62, P68, and P70) were related to DPPH free radical scavenging through the intersection of potentially active components in GRA, PLSR, and BP-ANN combined with MIV algorithm. Nine components (P4, P7, P10, P17, P37, P39, P46, P50, and P67) were related to FRAP results in the three chemometrics. Meanwhile, six components (P1, P4, P28, P37, P44, and P62) were relevant to inhibit the proliferation ability of A549 cells; twenty-two components (P2, P7, P8, P13, P20, P21, P23, P24, P28, P30, P34, P35, P36, P42, P44, P48, P49, P51, P60, P62, P68, and P70) were relevant to inhibit the horizontal migration ability of A549 cells; five components (P24, P28, P44, P62, and P70) and twelve components (P1, P5, P7, P10, P16, P21, P23, P24, P28, P34, P35, and P70) were respectively relevant to inhibit the vertical migration and invasion abilities of A549 cells.

### 3.5 Evaluation of potential active components

#### 3.5.1 Evaluation of antioxidant components

**FIGURE 7:**
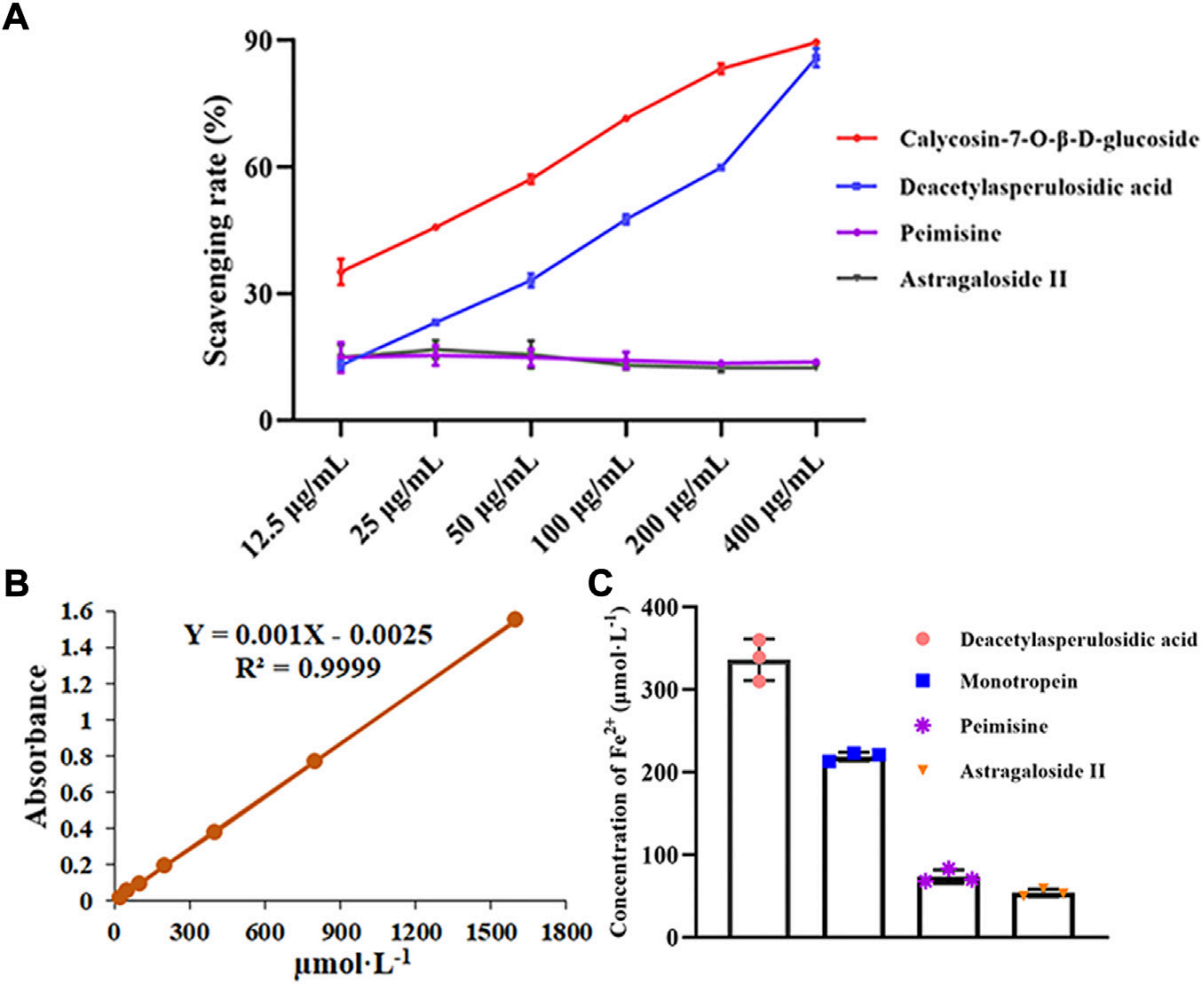
The antioxidant capacities of screened potentially active ingredients and unscreened ingredients were assessed (*n* = 3). **(A)** DPPH radical scavenging abilities of these components. **(B)** Standard curve of Fe^2+^. **(C)** The reducing power of these components. Data are represented as mean ± SD.

As shown in Figure 7A, the DPPH radical scavenging ability of calycosin-7-*O*-*β*-D-glucoside and deacetyl asperulosidic acid gradually increased with the increase of concentration, and their IC50 values were 30.24 µg mL^−1^ and 102.30 µg mL^−1^, respectively. However, peimine and astragaloside II had no significant change with the increase of concentration, showed weaker scavenging ability. And the scavenging rates ranged from 13.52% to 15.42% and 12.51%–16.76%, respectively.

As shown in Figure 7C, the FRAP values of deacetyl asperulosidic acid and monotropein were 336.33 and 219.00 µmol L^−1^, indicating strong reducing power. While peimine and astragaloside II showed relatively weak reducing power, and the FRAP values were 73.67 and 54.00 µmol L^−1^, respectively.

#### 3.5.2 Evaluation of potential active components for inhibiting cell viability

**FIGURE 8:**
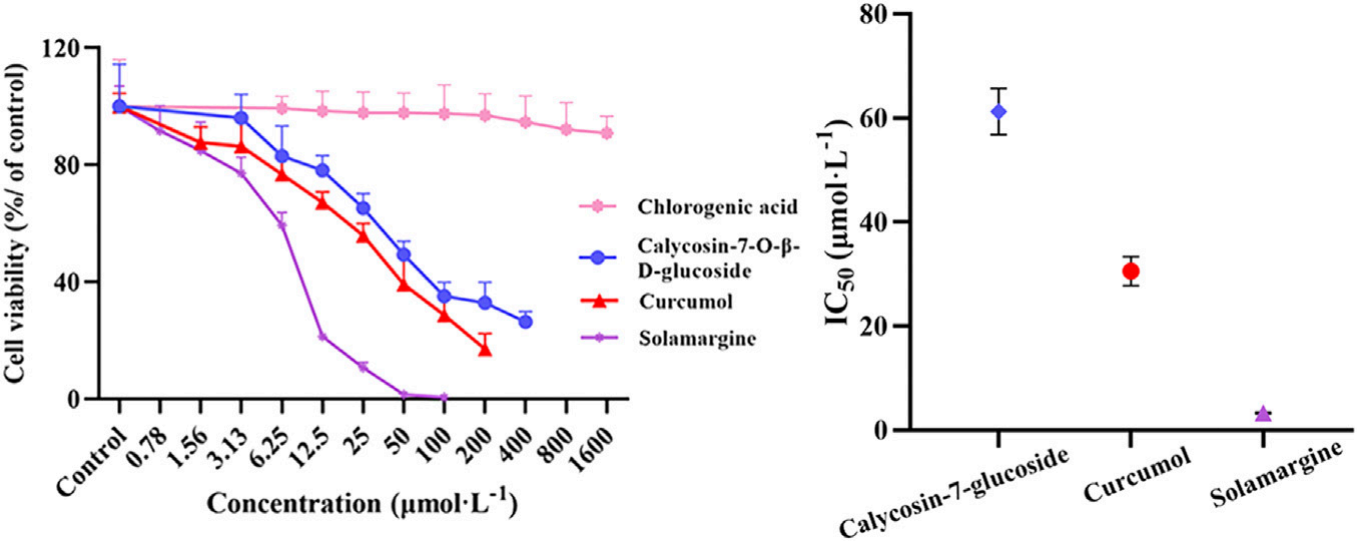
Effects of screened potentially active ingredients and unscreened ingredients on the proliferation of A549 cells were determined by CCK-8 kit (n = 6). Data are represented as mean ± SD.

As shown in Figure 8, the screened potentially active components (calycosin-7-*O*-*β*-D-glucoside, curcumol and solamargine) showed better inhibitory effects on A549 cell proliferation compared with the control group, and the IC50 values were 61.22, 30.56, and 3.29 µmol L^−1^, respectively. Whereas, chlorogenic acid had no significant change with the increase of concentration, showed weaker inhibitory effects. The cell survival rates ranged from 90.88% to 99.31%.

## 4 Discussion

QYSLD has been used to treat NSCLC for more than 20 years in the clinic, and its curative effect is definite. It has been confirmed that QYSLD can inhibit NSCLC by regulating the expression of PI3K/Akt/mTOR and Wnt/*β*-catenin signal transduction pathway molecules (Zhang and Li, 2017; Tong et al., 2018b). However, the active components information on the efficacy of QYSLD intervention in NSCLC has not yet been fully elucidated. Therefore, we established a fingerprint method based on UPLC-Q/TOF-MS to characterize the common peaks of ten batches of QYSLD. Combined with antioxidant and anti-NSCLC experiments, the spectrum-effect relationship analysis method was constructed to explore the main potentially active ingredients in QYSLD.

### 4.1 Analysis of antioxidant components of Qi-Yu-San-Long decoction

Previous studies have shown that oxidative stress plays a quite important role in tumor initiation, promotion and progression (Mileo and Miccadei, 2016). Moreover, cancer cells with accelerated metabolism require high ROS and free radical concentrations which are necessary to maintain their high propagation rate (Alkadi, 2020). At present, there are many methods for evaluating antioxidant effect, such as antiradical (DPPH and 2,2′-azino-bis(3-ethylbenzothiazoline) 6-sulfonic acid), phosphomolybdenum, reducing power (FRAP and cupric ion reducing activity), etc. Among them, DPPH free radical scavenging mainly involves the process of electron and H atom transfer (*via* direct reduction or radical quenching). Whereas, FRAP assay mainly involves the phenomenon of transferring an electron and reducing the TPTZ/ferric ion complex (antioxidants with a redox potential lower than 0.7 V) (Nunes et al., 2020).

In this study, DPPH-scavenging capacity and FRAP experiments were determined to evaluate different and complementary antioxidant activity mechanisms of QYSLD. DPPH radicals have a single-electron structure, which can extract an electron or atom from other substrates to induce the oxidative decomposition of substrates. While compounds containing phenolic hydroxyl groups can contribute H atom to inhibit free radical-induced oxidation. At the same time, there are some functional groups such as carboxyl groups, aliphatic hydroxyl groups, sulfur-related groups, imine groups, etc., which may also provide H atom or electron with the ability to scavenge free radicals (Liu, 2020). In this study, seventy common components and antioxidant effect were correlated by stoichiometric methods (GRA, PLSR, and BP-ANN combined with MIV algorithm), and found that L-arginine (P1), deacetyl asperulosidic acid (P4), heliosin (P24), calycosin-7-*O*-*β*-D-glucoside (P28), azelaic acid (P34), curcumol (P62), ursolic acid (P68) and linoleic acid (P70) were highly correlated with DPPH free radical scavenging. Among them, P4 and P28 were used for the evaluation experiment. Then, the results showed that P4 and P28 exhibited DPPH radical scavenging ability with IC50 values of 102.30 and 30.24 µg ml^−1^. Meanwhile, peimisine and astragaloside II, which failed to be selected as antioxidant ingredients, were chosen for parallel experiments. The scavenging rates ranged from 13.52% to 15.42% and 12.51%–16.76%, respectively. The results showed that they had poor ability to scavenge DPPH free radicals. It indicated that the eight components screened by spectrum-effect relationship had the exact effect of scavenging DPPH free radicals, which can be related to the presence of phenolic hydroxyl or carboxyl groups or aliphatic hydroxyl groups in their structure. These groups can provide H atom to scavenge DPPH free radicals. In addition, previous studies (Chen et al., 2005; Yu et al., 2005; Pânzariu et al., 2016; Somantri et al., 2021) have shown that L-arginine (P1), heliosin (P24), calycosin-7-*O*-*β*-D-glucoside (P28), ursolic acid (P68) had revealed great ability to scavenge DPPH free radicals.

The spectrum-effect results showed that deacetyl asperulosidic acid (P4), monotropein (P7), 3,8-diol-quinoline (P10), unknown component (P17), unknown component (P37), *E*-6-*O*-*p*-coumaroyl scandoside methyl ester (P39), solasodine (P46), zedoarol (P50) and linolenic acid (P67) have strong correlation with FRAP. Among them, deacetyl asperulosidic acid and monotropein were used for the evaluation experiment of iron ion reduction, and the FRAP values were 336.33 and 219.00 µmol L^−1^. At the same time, the FRAP values of peimisine and astragaloside II were 73.67 and 54.00 µmol L^−1^. It indicated that the nine components screened by the spectrum-effect relationship have the exact effect of inhibiting the reduction of iron ions, which may be related to their ability to transfer an electron and reduce the TPTZ/ferric ion complex. In these antioxidant ingredients, P4 was associated with both DPPH and FRAP, suggesting that P4 may greatly contribute to the antioxidant effect of QYSLD. According to published report (Ma et al., 2013), deacetyl asperulosidic acid (P4) may contribute to the antioxidant effect by increasing the activity of catalase and SOD.

### 4.2 Analysis of potential anti-non-small cell lung cancer active components

Metastasis is a complex multi-step process consisting tumor cell migration, intravasation, survival in the circulation, extravasation and proliferation within the distant organ parenchyma (Yang et al., 2019). In this work, CCK-8 assay was performed to determine the effect of A549 cells on proliferation, and wound healing and transwell assays were carried out to detect the metastatic ability of A549 cells. The spectrum-effect relationship between the seventy common peak areas and their pharmacodynamic indexes was constructed by GRA, PLSR and BP-ANN combined with MIV algorithm. According to the results of the spectrum-effect relationship, P1, P4, P28, P37, P44, and P62 played important roles in inhibiting the proliferation of A549 cells; P2, P7, P8, P13, P20, P21, P23, P24, P28, P30, P34, P35, P36, P42, P44, P48, P49, P51, P60, P62, P68, and P70 had important contributions in inhibiting the horizontal migration of A549 cells; P24, P28, P44, P62, and P70 were related to the inhibition of A549 cells vertical migration; P1, P5, P7, P10, P16, P21, P23, P24, P28, P34, P35, and P70 were related to the inhibition of A549 cells invasion. Subsequently, we assessed the three components (calycosin-7-*O*-*β*-D-glucoside, curcumol and solamargine) that were closely related to the inhibitory effect of A549 cell proliferation, and conducted parallel experiments on the component (chlorogenic acid) that was not closely related to this ‘effect’. With the increase of the concentration of calycosin-7-*O*-*β*-D-glucoside, curcumol and solamargine, the cell survival rates were inhibited with IC50 values of 61.21, 30.11 and 3.32 µmol L^−1^. And chlorogenic acid had a weak ability (the cell survival rates ranged from 90.88% to 99.31%) to inhibit A549 cell proliferation.

Calycosin-7-*O*-*β*-D-glucoside (P28) showed excellent efficacy results in inhibiting the proliferation, horizontal migration, vertical migration, and invasion abilities of A549 cells. It is worth mentioning that P28, an isoflavone compound, might be an effective component of HQ in the treatment of NSCLC, which has a therapeutic effect *via* autophagy and the p53/AMPK/mTOR signaling pathway (Yang et al., 2020). Heliosin (P24) and linoleic acid (P70) were regarded as candidate peaks in inhibiting the migration and invasion abilities of A549 cells. P24 is a quercetin glycoside that will release sugar groups during chewing, digestion and absorption. The metabolism of quercetin mainly involves the forms of glucuronidation, methylation and sulfate, which has potential significance in anti-inflammatory, antioxidant, and anticancer aspects (Tang et al., 2020). Solamargine (P44) and curcumol (P62) were regarded as candidate peaks in inhibiting the proliferation and migration abilities of A549 cells. P44 and P62 are derived from LK and EZ, respectively. According to previous reports, solamargine has been found to have anticancer activities and multi drug resistance by acting on a variety of biological pathways, including tumor suppressor pathways, mitochondrial pathways, death receptor pathways and cell survival pathways (Kalalinia and Karimi-Sani, 2017). Curcumol has been proven to be an effective inducer of apoptosis in many cancer cells by targeting key signaling pathways, such as MAPK/ERK, PI3K/Akt and NF-κB, suggesting its multi-target activity in anticancer therapy. It suggests that P44 and P62 are of great importance as anticancer drugs in future (Wei et al., 2019).

In summary, we intersected the potential active ingredients of antioxidant and anti-NSCLC activities (DPPH, FRAP, cell viability, wound healing, cell migration and cell invasion) and found that no component in QYSLD could be related to the six indexes at the same time. However, there are intersecting potentially active ingredients among each experiment. P1, P4, P28, and P62 were associated with DPPH free radical scavenging and cell viability, and P7 was associated with FRAP, wound healing and transwell invasion. It is suggested that there is a partial relationship between antioxidant and anti-tumor, and it also implied that these shared potentially active components may provide a greater contribution to the therapeutic effect of QYSLD.

## 5 Conclusion

In this study, a fingerprint method based on UPLC-Q/TOF-MS was successfully established to obtain common peaks of ten batches of QYSLD. To explore the main potentially active ingredients in QYSLD, the common peak areas and antioxidant or anti-NSCLC activities were used to establish the spectrum-effect relationship *via* GRA, PLSR, and BP-ANN combined with MIV algorithm. In terms of antioxidant effect, eight ingredients (L-arginine (P1), deacetyl asperulosidic acid (P4), heliosin (P24), etc.) of QYSLD were relevant to DPPH free radical scavenging ability; nine ingredients (deacetyl asperulosidic acid (P4), monotropein (P7), *E*-6-*O*-*p*-coumaroyl scandoside methyl ester (P39), etc.) were relevant to FRAP. In terms of anti-NSCLC activity, six ingredients (L-arginine (P1), deacetyl asperulosidic acid (P4), calycosin-7-*O*-*β*-D-glucoside (P28), etc.) were relevant to inhibit the proliferation ability of A549 cells; twenty-two ingredients (monotropein (P7), caffeic acid (P20), calycosin-7-*O*-*β*-D-glucoside (P28), etc.) were considered as candidate ingredients for inhibiting the horizontal migration ability of A549 cells; five ingredients (heliosin (P24), calycosin-7-*O*-*β*-D-glucoside (P28), solamargine (P44), etc.) were considered as candidate ingredients for inhibiting the vertical migration ability of A549 cells; twelve ingredients (L-arginine (P1), monotropein (P7), asperulosidic acid (P16), etc.) were considered as candidate ingredients for inhibiting the invasion ability of A549 cells. Among them, parts of candidate ingredients, which were predicted by the chemometrics, were assessed through confirmatory experiments. Compared with the chemical components that were not selected as active ingredients, the results indicated that the screened potentially active ingredients showed better antioxidant and inhibitory effects on A549 cell proliferation. In general, this study found the potential active ingredients in QYSLD, and provided reference for the discovery of active ingredients of TCM.

## Data Availability

The original contributions presented in the study are included in the article/Supplementary Material, further inquiries can be directed to the corresponding author.

## Glossary

BHSSC: Hedyotis diffusa Willd. (Baihuasheshecao)
BP-ANN: backpropagation neural network
CBM: Fritillaria cirrhosa D.Don (Chuanbeimu)
CV: coefficient of variance
DL: Pheretima aspergillum (E. Perrier) (Dilong)
DMEM: Dulbecco’s modified of Eagle’s medium
DPPH: 1,1-Diphenyl-2-picrylhydrazyl radical
ESI: electrospray ionization
EZ: Curcuma phaeocaulis Valeton (Ezhu)
FBS: fetal bovine serum
GRA: grey correlation analysis
HQ: Astragalus mongholicus Bunge (Huangqi)
HRMS: high-resolution mass spectrometry
IC50: 50% inhibitory concentration
IS: internal standard
LC-ELSD: liquid chromatography-evaporative light scattering detector
LC-UV: liquid chromatography-ultraviolet detection
LK: Solanum nigrum L. (Longkui)
MIV: mean impact value
NSCLC: non-small cell lung cancer
OD: optical density
PBS: phosphate buffer saline
PLSR: partial least squares regression
QC: quality control
QYSLD: Qi-Yu-San-Long decoction
ROS: reactive oxygen species
RPA: relative peak area
RRT: relative retention time
RSD: relative standard deviation
SCLC: small cell lung cancer
TCM: traditional Chinese medicine
TL: Scolopendra subspinipes mutilans L.Koch (Tianlong)
TPTZ: 2,4,6-Tris(2-pyridyl)-s-triazine
UPLC-Q-TOF/MS: ultra-high liquid chromatography-quadrupole-time-of-flight mass spectrometry
VIP: variable influence on projection
YYR: Coix lacryma-jobi L. (Yiyiren)
YZ: Polygonatum odoratum (Mill.) Druce (Yuzhu)
ZQ: Euphorbia helioscopia L. (Zeqi)
