# Supplementary material for: Discovering the potential active ingredients of Qi-Yu-San-Long decoction for anti-oxidation, inhibition of non-small cell lung cancer based on the spectrum-effect relationship combined with chemometric methods
Source: Front Pharmacol. 2022 Oct 19;13:989139. doi: 10.3389/fphar.2022.989139 (PMC9627220; doi:10.3389/fphar.2022.989139)
Supplement: Supplementary file 1 [file DataSheet1.docx]

***Supplementary Material***

**Discovering the potential active ingredients of Qi-Yu-San-Long decoction for anti-oxidation, inhibition of non-small cell lung cancer based on the spectrum-effect relationship combined with chemometric methods**

Mengwen Huang ^1^, Ruijuan Li ^1^, Mo Yang ^1^, An Zhou ^1^, Hong Wu ^2^, Zegeng Li ^1,2,3^, Huan Wu ^1,2 *^

^1^ *Key Laboratory of Xin'an Medicine, Ministry of Education,* *Anhui University of Chinese Medicine, Hefei, 230038, China*

^2^ *Anhui Province Key Laboratory of Chinese Medicinal Formula & Anhui Province Key Laboratory of Research and Development of Chinese Medicine, Hefei, 230012, China*

^3^ *Key Laboratory of Traditional Chinese medicine for Prevention and Treatment of Major Pulmonary Diseases, Department of Education of Anhui Province, Hefei, 230038, China*

^∗^ Corresponding author at: Anhui University of Chinese Medicine, Meishan Road No.103, Hefei 230038, China. E-mail address: [wuhuancpu@163.com](mailto:wuhuancpu@163.com) (H. Wu).

**Tables**

**Table S1** Detailed information of the botanical and animal drugs composed in QYSLD

**Table S2** Orthogonal experiment on preparation technology of QYSLD

**Table S3** Method evaluation results of UPLC-Q/TOF-MS fingerprints analysis

**Table S4** Similarity evaluation results of ten batches of QYSLD samples

**Table S5** Relative retention time of common peaks in ten batches of QYSLD

**Table S6** Relative peak areas of common peaks in ten batches of QYSLD

**Table S7** MS data and identification results of common peaks of QYSLD

**Table S8** Correlations and grade of GRA between common peaks and the antioxidant, anti-NSCLC activities, respectively

**Table S9** Correlation coefficient and VIP values of PLSR between common peaks and the antioxidant, anti-NSCLC activities, respectively

**Table S10** Correlations and grade of MIV values between common peaks and the antioxidant, anti-NSCLC activities, respectively

**Table S1**

Detailed information of the botanical and animal drugs composed in QYSLD

| Chinese name | Latin name | Family | Origin | Lot number | Manufacturer |
| --- | --- | --- | --- | --- | --- |
| Huangqi | *Astragalus mongholicus* Bunge | Fabaceae | Dingxi, China | 200903 | Bozhou Jingwan Chinese Herbal Pieces Factory |
| Longkui | *Solanum nigrum* L. | Solanaceae | Bozhou, China | 200201 | Bozhou Jingwan Chinese Herbal Pieces Factory |
| Baihuasheshecao | *Hedyotis diffusa* Willd. | Rubiaceae | Fuzhou, China | 200301 | Bozhou Jingwan Chinese Herbal Pieces Factory |
| Yiyiren | *Coix lacryma-jobi* L. | Poaceae | Xingyi, China | 200601 | Bozhou Jingwan Chinese Herbal Pieces Factory |
| Ezhu | *Curcuma phaeocaulis* Valeton | Zingiberaceae | Nanning, China | 191101 | Bozhou Jingwan Chinese Herbal Pieces Factory |
| Yuzhu | *Polygonatum odoratum* (Mill.) Druce | Asparagaceae | Shaoyang, China | 191101 | Bozhou Jingwan Chinese Herbal Pieces Factory |
| Tianlong | *Scolopendra subspinipes mutilans* L.Koch | Scolopendridae | Jiangsu, China | 181125001 | Beijing Meikantang Medical Technology Co., LTD |
| Dilong | *Pheretima aspergillum* (E. Perrier) | Megascolecidae | Guangdong, China | 151001 | Bozhou Jingwan Chinese Herbal Pieces Factory |
| Zeqi | *Euphorbia helioscopia* L. | Euphorbiaceae | Anhui, China | 171001 | Bozhou Jingwan Chinese Herbal Pieces Factory |
| Chuanbeimu | *Fritillaria cirrhosa* D.Don | Liliaceae | Sichuan, China | 141001 | Bozhou Jingwan Chinese Herbal Pieces Factory |

**Table S2**

Orthogonal experiment on preparation technology of QYSLD

| Number | Solid-liquid ratio | Soaking time | Boiling time | Decocting times |
| --- | --- | --- | --- | --- |
| S1 | 1:8 | 0.5 h | 0.5 h | 1 |
| S2 | 1:8 | 1 h | 1 h | 2 |
| S3 | 1:8 | 1.5 h | 1.5 h | 3 |
| S4 | 1:10 | 0.5 h | 1 h | 3 |
| S5 | 1:10 | 1 h | 1.5 h | 1 |
| S6 | 1:10 | 1.5 h | 0.5 h | 2 |
| S7 | 1:12 | 0.5 h | 1.5 h | 2 |
| S8 | 1:12 | 1 h | 0.5 h | 3 |
| S9 | 1:12 | 1.5 h | 1 h | 1 |

**Table S3**

Method evaluation results of UPLC-Q/TOF-MS fingerprints analysis

| Peak No. | Linear regression | R^2^ | Linear range (μ*g*·mL^-1^) | Precision  (RSD, %) | | Repeatability  (RSD, %) | | Stability (24 h)  (RSD, %) | |
| --- | --- | --- | --- | --- | --- | --- | --- | --- | --- |
|  |  |  |  | **RRT** | **RRA** | **RRT** | **RRA** | **RRT** | **RRA** |
| P1 | Y = 0.0147X + 0.6256 | 0.9938 | 0.53-6.48 | 0.32% | 5.47% | 0.76% | 4.75% | 0.76% | 5.39% |
| P2 | Y = 0.0679X - 0.5523 | 0.9920 | 0.44-25.90 | 0.28% | 5.65% | 0.22% | 6.60% | 0.41% | 4.38% |
| P3 | Y = 0.0334X - 0.5921 | 0.9743 | 0.26-12.41 | 0.91% | 6.90% | 0.83% | 7.57% | 0.96% | 8.14% |
| P4 | Y = 0.0375X - 0.0208 | 0.9962 | 0.41-15.24 | 0.81% | 5.29% | 0.81% | 6.72% | 0.81% | 6.01% |
| P5 | Y = 0.0249X - 0.5138 | 0.9911 | 0.14-9.69 | 0.63% | 3.63% | 0.39% | 4.18% | 0.15% | 5.15% |
| P6 | Y = 0.1257X + 0.4519 | 0.9910 | 0.48-49.32 | 0.64% | 6.30% | 0.47% | 6.23% | 0.72% | 7.22% |
| P7 | Y = 0.0583X - 0.6012 | 0.9976 | 0.33-22.70 | 0.71% | 5.82% | 0.72% | 6.37% | 0.59% | 6.51% |
| P8 | Y = 0.1509X - 2.0036 | 0.9994 | 0.14-58.09 | 0.58% | 3.72% | 0.63% | 4.84% | 1.02% | 3.68% |
| P9 | Y = 0.0475X - 0.5308 | 0.9927 | 0.15-18.01 | 0.29% | 5.98% | 0.12% | 6.40% | 0.19% | 4.58% |
| P10 | Y = 0.1513X - 3.7952 | 0.9925 | 0.10-56.20 | 0.31% | 4.76% | 0.88% | 6.52% | 0.72% | 6.02% |
| P11 | Y = 0.1063X - 2.9749 | 0.9833 | 0.15-41.45 | 0.58% | 7.35% | 0.64% | 7.87% | 0.63% | 7.96% |
| P12 | Y = 0.0912X - 1.8359 | 0.9967 | 0.22-34.63 | 0.24% | 6.61% | 0.51% | 6.11% | 0.15% | 5.19% |
| P13 | Y = 0.0343X - 0.9312 | 0.9918 | 0.02-13.20 | 0.27% | 3.80% | 0.34% | 5.63% | 0.36% | 4.92% |
| P14 | Y = 0.0445X - 0.6492 | 0.9954 | 0.15-16.99 | 0.20% | 6.98% | 0.55% | 2.67% | 0.19% | 6.92% |
| P15 | Y = 0.0070X - 0.1086 | 0.9930 | 0.05-2.71 | 0.00% | 7.13% | 0.00% | 6.52% | 0.00% | 4.17% |
| P16 | Y = 0.0793X - 1.4314 | 0.9967 | 0.31-30.55 | 0.25% | 6.83% | 0.22% | 7.08% | 0.21% | 7.80% |
| P17 | Y = 0.0228X - 0.7039 | 0.9909 | 0.02-8.60 | 0.10% | 4.73% | 0.04% | 7.28% | 0.05% | 7.85% |
| P18 | Y = 0.0059X - 0.1241 | 0.9945 | 0.03-2.28 | 0.23% | 6.97% | 0.57% | 6.31% | 0.79% | 6.51% |
| P19 | Y = 0.0030X - 0.0679 | 0.9934 | 0.01-1.13 | 0.29% | 6.52% | 0.30% | 7.20% | 0.27% | 6.04% |
| P20 | Y = 0.0038X - 0.0524 | 0.9929 | 0.02-1.43 | 0.31% | 7.08% | 0.87% | 6.83% | 1.01% | 5.13% |
| P21 | Y = 0.0236X - 0.4391 | 0.9929 | 0.03-8.94 | 0.27% | 6.18% | 0.64% | 6.87% | 0.17% | 6.08% |
| P22 | Y = 0.0551X - 1.0966 | 0.9935 | 0.09-21.27 | 0.09% | 5.90% | 0.25% | 6.21% | 0.41% | 4.77% |
| P23 | Y = 0.0221X - 0.5434 | 0.9904 | 0.03-8.31 | 0.24% | 7.04% | 0.19% | 5.08% | 0.18% | 7.59% |
| P24 | Y = 0.0925X - 1.9881 | 0.9909 | 0.27-36.22 | 0.18% | 5.80% | 0.16% | 4.78% | 0.17% | 6.88% |
| P25 | Y = 0.0481X - 0.6296 | 0.9920 | 0.21-19.03 | 0.05% | 5.22% | 0.13% | 6.54% | 0.12% | 7.53% |
| P26 | Y = 0.0037X - 0.0601 | 0.9926 | 0.02-1.42 | 0.19% | 6.71% | 0.42% | 7.06% | 0.53% | 6.82% |
| P27 | Y = 0.0053X - 0.0896 | 0.9779 | 0.01-1.97 | 0.15% | 7.36% | 0.12% | 6.88% | 0.15% | 7.88% |
| P28 | Y = 0.0546X - 0.8337 | 0.9985 | 0.22-21.15 | 0.09% | 6.54% | 0.10% | 4.65% | 0.08% | 5.57% |
| P29 | Y = 0.0524X - 0.8274 | 0.9987 | 0.20-20.35 | 0.07% | 5.36% | 0.04% | 6.89% | 0.09% | 6.34% |
| P30 | Y = 0.0341X - 0.6063 | 0.9976 | 0.09-13.11 | 0.06% | 7.52% | 0.05% | 5.51% | 0.09% | 7.47% |
| P31 | Y = 0.0578X - 1.4790 | 0.9813 | 0.17-22.77 | 0.03% | 6.92% | 0.04% | 2.87% | 0.07% | 6.69% |
| P32 | Y = 0.0306X - 0.9021 | 0.9909 | 0.03-11.67 | 0.15% | 4.58% | 0.39% | 6.49% | 0.59% | 6.88% |
| P33 | Y = 0.1035X - 2.6598 | 0.9753 | 0.33-41.02 | 0.04% | 6.97% | 0.03% | 3.38% | 0.09% | 8.19% |
| P34 | Y = 0.0323X - 0.8130 | 0.9915 | 0.05-12.52 | 0.03% | 4.19% | 0.04% | 7.71% | 0.06% | 4.37% |
| P35 | Y = 0.1269X - 1.7122 | 0.9938 | 0.24-48.03 | 0.72% | 6.23% | 0.28% | 3.84% | 0.25% | 7.22% |
| P36 | Y = 0.1442X + 5.1248 | 0.9904 | 4.17-61.20 | 0.04% | 7.32% | 0.03% | 6.61% | 0.06% | 7.39% |
| P37 | Y = 0.1009X - 2.0249 | 0.9916 | 0.52-39.27 | 0.59% | 5.98% | 0.12% | 7.82% | 0.15% | 6.32% |
| P38 | Y = 0.0898X - 2.5493 | 0.9917 | 0.09-33.91 | 0.15% | 4.97% | 0.11% | 5.44% | 0.17% | 6.36% |
| P39 | Y = 0.0785X - 1.9744 | 0.9926 | 0.19-30.23 | 0.04% | 6.12% | 0.05% | 3.30% | 0.07% | 6.38% |
| P40 | Y = 0.0315X - 0.7881 | 0.9835 | 0.14-12.36 | 0.03% | 6.50% | 0.00% | 7.33% | 0.06% | 5.80% |
| P41 | Y = 0.1056X - 4.7951 | 0.9116 | 0.14-42.03 | 0.04% | 7.32% | 0.08% | 3.70% | 0.08% | 7.17% |
| P42 | Y = 0.1040X - 2.2350 | 0.9957 | 0.21-39.69 | 0.11% | 4.86% | 0.31% | 6.19% | 0.37% | 7.50% |
| P43 | Y = 0.0390X - 0.5654 | 0.9976 | 0.17-15.06 | 0.02% | 7.05% | 0.07% | 4.80% | 0.05% | 5.24% |
| P44 | Y = 0.1840X - 0.0999 | 0.9966 | 0.75-72.55 | 0.11% | 6.32% | 0.29% | 5.51% | 0.35% | 5.32% |
| P45 | Y = 0.0925X - 2.1070 | 0.9770 | 0.56-36.83 | 0.05% | 6.67% | 0.07% | 2.70% | 0.08% | 6.58% |
| P46 | Y = 0.0279X - 0.7345 | 0.9922 | 0.06-10.75 | 0.51% | 6.70% | 0.43% | 5.62% | 0.42% | 6.07% |
| P47 | Y = 0.1963X + 0.6448 | 0.9927 | 0.88-78.97 | 0.31% | 5.38% | 0.21% | 7.62% | 0.24% | 7.69% |
| P48 | Y = 0.0496X - 1.3454 | 0.9910 | 0.07-19.02 | 0.05% | 6.88% | 0.24% | 2.03% | 0.05% | 7.00% |
| P49 | Y = 0.0564X - 0.8488 | 0.9984 | 0.20-21.95 | 0.03% | 7.72% | 0.24% | 4.86% | 0.03% | 6.22% |
| P50 | Y = 0.0071X - 0.0355 | 0.9954 | 0.10-2.88 | 0.15% | 6.86% | 0.12% | 6.09% | 0.13% | 7.40% |
| P51 | Y = 0.0942X - 0.9289 | 0.9959 | 0.56-36.21 | 0.05% | 5.83% | 0.07% | 6.89% | 0.06% | 5.58% |
| P52 | Y = 0.1943X - 4.9203 | 0.9925 | 0.06-72.07 | 0.07% | 6.83% | 0.18% | 7.30% | 0.31% | 6.83% |
| P53 | Y = 0.0599X - 1.9703 | 0.9537 | 0.13-23.83 | 0.03% | 4.91% | 0.05% | 6.64% | 0.04% | 7.48% |
| P54 | Y = 0.1221X - 2.2459 | 0.9934 | 0.61-47.89 | 0.03% | 5.92% | 0.04% | 3.96% | 0.03% | 6.66% |
| P55 | Y = 0.0440X - 0.9805 | 0.9943 | 0.14-16.67 | 0.02% | 6.26% | 0.04% | 5.80% | 0.03% | 6.87% |
| P56 | Y = 0.0187X - 0.4882 | 0.9922 | 0.04-7.19 | 0.04% | 5.77% | 0.04% | 6.34% | 0.04% | 6.42% |
| P57 | Y = 0.2002X + 11.2330 | 0.9635 | 5.02-87.80 | 0.03% | 6.17% | 0.06% | 6.15% | 0.04% | 5.12% |
| P58 | Y = 0.2154X - 3.1300 | 0.9937 | 1.04-84.84 | 0.01% | 6.56% | 0.03% | 5.26% | 0.03% | 6.19% |
| P59 | Y = 0.1335X - 1.9061 | 0.9959 | 0.58-52.00 | 0.02% | 6.36% | 0.03% | 4.20% | 0.03% | 6.49% |
| P60 | Y = 0.1225X + 3.0884 | 0.9913 | 2.89-50.81 | 0.03% | 4.17% | 0.04% | 5.01% | 0.04% | 3.90% |
| P61 | Y = 0.0585X - 1.5514 | 0.9905 | 0.10-21.84 | 0.02% | 7.11% | 0.05% | 4.44% | 0.04% | 5.59% |
| P62 | Y = 0.0617X - 1.1687 | 0.9972 | 0.54-11.29 | 0.02% | 5.26% | 0.04% | 4.24% | 0.03% | 5.71% |
| P63 | Y = 0.1860X - 0.8012 | 0.9926 | 0.86-71.62 | 0.06% | 6.48% | 0.05% | 2.93% | 0.05% | 7.17% |
| P64 | Y = 0.0503X - 0.4783 | 0.9915 | 0.12-19.73 | 0.09% | 6.82% | 0.06% | 5.22% | 0.08% | 6.08% |
| P65 | Y = 0.1973X + 0.0126 | 0.9965 | 0.76-77.61 | 0.28% | 5.51% | 0.05% | 6.51% | 0.04% | 4.13% |
| P66 | Y = 0.1508X - 3.8578 | 0.9912 | 0.34-58.28 | 0.01% | 7.32% | 0.17% | 5.00% | 0.09% | 7.70% |
| P67 | Y = 0.0518X - 1.1955 | 0.9919 | 0.19-20.15 | 0.02% | 6.30% | 0.04% | 6.20% | 0.02% | 5.27% |
| P68 | Y = 0.0933X - 0.5327 | 0.9971 | 0.85-37.19 | 0.14% | 5.85% | 0.06% | 5.43% | 0.14% | 7.42% |
| P69 | Y = 0.0481X + 1.7014 | 0.9845 | 1.16-20.32 | 0.17% | 6.60% | 0.04% | 4.98% | 0.07% | 8.17% |
| P70 | Y = 0.1174X - 1.8767 | 0.9909 | 0.92-46.06 | 0.02% | 5.82% | 0.07% | 4.94% | 0.01% | 5.70% |

**Table S4**

Similarity evaluation results of ten batches of QYSLD samples

| ESI^+^ |  |  |  |  |  |  |  |  |  |  |  |
| --- | --- | --- | --- | --- | --- | --- | --- | --- | --- | --- | --- |
|  | S1 | S2 | S3 | S4 | S5 | S6 | S7 | S8 | S9 | S10 | R |
| S1 | 1.000 | 0.871 | 0.849 | 0.826 | 0.841 | 0.825 | 0.830 | 0.801 | 0.830 | 0.774 | 0.882 |
| S2 | 0.871 | 1.000 | 0.927 | 0.895 | 0.923 | 0.918 | 0.898 | 0.869 | 0.909 | 0.844 | 0.950 |
| S3 | 0.849 | 0.927 | 1.000 | 0.923 | 0.899 | 0.905 | 0.887 | 0.868 | 0.877 | 0.862 | 0.945 |
| S4 | 0.826 | 0.895 | 0.923 | 1.000 | 0.912 | 0.906 | 0.892 | 0.865 | 0.872 | 0.894 | 0.945 |
| S5 | 0.841 | 0.923 | 0.899 | 0.912 | 1.000 | 0.950 | 0.953 | 0.900 | 0.965 | 0.884 | 0.971 |
| S6 | 0.825 | 0.918 | 0.905 | 0.906 | 0.950 | 1.000 | 0.952 | 0.921 | 0.941 | 0.888 | 0.970 |
| S7 | 0.830 | 0.898 | 0.887 | 0.892 | 0.953 | 0.952 | 1.000 | 0.936 | 0.962 | 0.925 | 0.974 |
| S8 | 0.801 | 0.869 | 0.868 | 0.865 | 0.900 | 0.921 | 0.936 | 1.000 | 0.933 | 0.929 | 0.953 |
| S9 | 0.830 | 0.909 | 0.877 | 0.872 | 0.965 | 0.941 | 0.962 | 0.933 | 1.000 | 0.913 | 0.970 |
| S10 | 0.774 | 0.844 | 0.862 | 0.894 | 0.884 | 0.888 | 0.925 | 0.929 | 0.913 | 1.000 | 0.942 |
| R | 0.882 | 0.950 | 0.945 | 0.945 | 0.971 | 0.970 | 0.974 | 0.953 | 0.970 | 0.942 | 1.000 |
| ESI^−^ |  |  |  |  |  |  |  |  |  |  |  |
|  | S1 | S2 | S3 | S4 | S5 | S6 | S7 | S8 | S9 | S10 | R |
| S1 | 1.000 | 0.871 | 0.644 | 0.716 | 0.591 | 0.764 | 0.653 | 0.701 | 0.697 | 0.552 | 0.797 |
| S2 | 0.871 | 1.000 | 0.872 | 0.645 | 0.716 | 0.591 | 0.764 | 0.660 | 0.701 | 0.702 | 0.812 |
| S3 | 0.644 | 0.872 | 1.000 | 0.735 | 0.756 | 0.624 | 0.840 | 0.700 | 0.835 | 0.805 | 0.861 |
| S4 | 0.716 | 0.645 | 0.735 | 1.000 | 0.827 | 0.933 | 0.915 | 0.919 | 0.874 | 0.966 | 0.936 |
| S5 | 0.591 | 0.716 | 0.756 | 0.827 | 1.000 | 0.849 | 0.912 | 0.915 | 0.924 | 0.891 | 0.933 |
| S6 | 0.764 | 0.591 | 0.624 | 0.933 | 0.849 | 1.000 | 0.872 | 0.962 | 0.855 | 0.909 | 0.913 |
| S7 | 0.653 | 0.764 | 0.840 | 0.915 | 0.912 | 0.872 | 1.000 | 0.907 | 0.913 | 0.940 | 0.962 |
| S8 | 0.701 | 0.660 | 0.700 | 0.919 | 0.915 | 0.962 | 0.907 | 1.000 | 0.908 | 0.928 | 0.953 |
| S9 | 0.697 | 0.701 | 0.835 | 0.874 | 0.924 | 0.855 | 0.913 | 0.908 | 1.000 | 0.921 | 0.950 |
| S10 | 0.552 | 0.702 | 0.805 | 0.966 | 0.891 | 0.909 | 0.940 | 0.928 | 0.921 | 1.000 | 0.967 |
| R | 0.797 | 0.812 | 0.861 | 0.936 | 0.933 | 0.913 | 0.962 | 0.953 | 0.950 | 0.967 | 1.000 |

**Table S5**

Relative retention time of common peaks in ten batches of QYSLD

| ESI^+^  Peaks | Samples |  |  |  |  |  |  |  |  |  |  |
| --- | --- | --- | --- | --- | --- | --- | --- | --- | --- | --- | --- |
|  | S1 | S2 | S3 | S4 | S5 | S6 | S7 | S8 | S9 | S10 | CV% |
| P1 | 0.0438 | 0.0445 | 0.0445 | 0.0438 | 0.0445 | 0.0438 | 0.0445 | 0.0438 | 0.0438 | 0.0438 | 0.84% |
| P2 | 0.0637 | 0.0637 | 0.0637 | 0.0634 | 0.0641 | 0.0634 | 0.0641 | 0.0634 | 0.0641 | 0.0634 | 0.49% |
| P3 | 0.0743 | 0.0743 | 0.0743 | 0.0736 | 0.0736 | 0.0732 | 0.0732 | 0.0724 | 0.0724 | 0.0717 | 1.17% |
| P4 | 0.0870 | 0.0870 | 0.0870 | 0.0870 | 0.0870 | 0.0870 | 0.0867 | 0.0870 | 0.0870 | 0.0870 | 0.13% |
| P5 | 0.0972 | 0.0965 | 0.0965 | 0.0965 | 0.0965 | 0.0972 | 0.0972 | 0.0972 | 0.0972 | 0.0965 | 0.39% |
| P6 | 0.0980 | 0.0980 | 0.0980 | 0.0981 | 0.0988 | 0.0947 | 0.0954 | 0.0966 | 0.0973 | 0.0973 | 1.27% |
| P7 | 0.1372 | 0.1372 | 0.1372 | 0.1372 | 0.1379 | 0.1379 | 0.1375 | 0.1379 | 0.1379 | 0.1379 | 0.26% |
| P8 | 0.1418 | 0.1418 | 0.1411 | 0.1403 | 0.1403 | 0.1399 | 0.1392 | 0.1381 | 0.1384 | 0.1381 | 0.96% |
| P9 | 0.2321 | 0.2321 | 0.2321 | 0.2329 | 0.2329 | 0.2329 | 0.2325 | 0.2329 | 0.2329 | 0.2329 | 0.15% |
| P10 | 0.2414 | 0.2380 | 0.2376 | 0.2433 | 0.2433 | 0.2414 | 0.2403 | 0.2380 | 0.2380 | 0.2376 | 0.92% |
| P11 | 0.2478 | 0.2466 | 0.2453 | 0.2516 | 0.2531 | 0.2550 | 0.2561 | 0.2588 | 0.2595 | 0.2588 | 1.98% |
| P12 | 0.2894 | 0.2894 | 0.2894 | 0.2901 | 0.2901 | 0.2901 | 0.2905 | 0.2905 | 0.2905 | 0.2905 | 0.16% |
| P13 | 0.3060 | 0.3060 | 0.3052 | 0.3052 | 0.3052 | 0.3052 | 0.3044 | 0.3048 | 0.3048 | 0.3048 | 0.15% |
| P14 | 0.3318 | 0.3279 | 0.3312 | 0.3320 | 0.3320 | 0.3320 | 0.3316 | 0.3320 | 0.3320 | 0.3320 | 0.36% |
| P15 | 0.3335 | 0.3341 | 0.3342 | 0.3342 | 0.3350 | 0.3342 | 0.3342 | 0.3342 | 0.3342 | 0.3342 | 0.10% |
| P16 | 0.3399 | 0.3399 | 0.3399 | 0.3406 | 0.3399 | 0.3406 | 0.3406 | 0.3406 | 0.3406 | 0.3406 | 0.11% |
| P17 | 0.3421 | 0.3421 | 0.3421 | 0.3429 | 0.3429 | 0.3429 | 0.3436 | 0.3429 | 0.3436 | 0.3436 | 0.17% |
| P18 | 0.3559 | 0.3559 | 0.3553 | 0.3527 | 0.3553 | 0.3519 | 0.3516 | 0.3519 | 0.3516 | 0.3527 | 0.50% |
| P19 | 0.3749 | 0.3755 | 0.3755 | 0.3606 | 0.3613 | 0.3606 | 0.3613 | 0.3613 | 0.3613 | 0.3613 | 1.78% |
| P20 | 0.4031 | 0.4037 | 0.4037 | 0.4013 | 0.4013 | 0.4013 | 0.3998 | 0.4005 | 0.4005 | 0.4005 | 0.34% |
| P21 | 0.4160 | 0.4160 | 0.4160 | 0.4164 | 0.4164 | 0.4164 | 0.4164 | 0.4164 | 0.4164 | 0.4164 | 0.04% |
| P22 | 0.4647 | 0.4647 | 0.4647 | 0.4640 | 0.4632 | 0.4632 | 0.4628 | 0.4621 | 0.4621 | 0.4621 | 0.23% |
| P23 | 0.5541 | 0.5535 | 0.5543 | 0.5543 | 0.5546 | 0.5546 | 0.5546 | 0.5543 | 0.5546 | 0.5543 | 0.06% |
| P24 | 0.5927 | 0.5927 | 0.5927 | 0.5934 | 0.5934 | 0.5934 | 0.5934 | 0.5934 | 0.5934 | 0.5938 | 0.07% |
| P25 | 0.6035 | 0.6035 | 0.6039 | 0.5971 | 0.5979 | 0.5979 | 0.5979 | 0.5986 | 0.5979 | 0.5986 | 0.44% |
| P26 | 0.6131 | 0.6125 | 0.6138 | 0.6138 | 0.6130 | 0.6138 | 0.6138 | 0.6161 | 0.6149 | 0.6142 | 0.16% |
| P27 | 0.6543 | 0.6543 | 0.6541 | 0.6364 | 0.6413 | 0.6353 | 0.6364 | 0.6356 | 0.6353 | 0.6353 | 1.29% |
| P28 | 0.7064 | 0.7070 | 0.7069 | 0.7076 | 0.7069 | 0.7076 | 0.7076 | 0.7076 | 0.7076 | 0.7076 | 0.06% |
| P29 | 0.7366 | 0.7372 | 0.7370 | 0.7374 | 0.7370 | 0.7370 | 0.7378 | 0.7378 | 0.7378 | 0.7378 | 0.05% |
| P30 | 0.7828 | 0.7835 | 0.7830 | 0.7833 | 0.7833 | 0.7833 | 0.7833 | 0.7841 | 0.7841 | 0.7841 | 0.06% |
| P31 | 0.8246 | 0.8248 | 0.8248 | 0.8252 | 0.8252 | 0.8252 | 0.8252 | 0.8259 | 0.8252 | 0.8259 | 0.05% |
| P32 | 0.8386 | 0.8389 | 0.8382 | 0.8276 | 0.8280 | 0.8280 | 0.8291 | 0.8291 | 0.8291 | 0.8280 | 0.56% |
| P33 | 0.8549 | 0.8549 | 0.8549 | 0.8549 | 0.8549 | 0.8549 | 0.8553 | 0.8553 | 0.8553 | 0.8553 | 0.02% |
| P34 | 0.8681 | 0.8681 | 0.8681 | 0.8681 | 0.8681 | 0.8681 | 0.8689 | 0.8689 | 0.8689 | 0.8689 | 0.04% |
| P35 | 0.9023 | 0.9027 | 0.9027 | 0.9023 | 0.9015 | 0.9023 | 0.9000 | 0.9000 | 0.8989 | 0.8982 | 0.18% |
| P36 | 0.9054 | 0.9062 | 0.9062 | 0.9062 | 0.9062 | 0.9062 | 0.9058 | 0.9062 | 0.9066 | 0.9066 | 0.03% |
| P37 | 0.9027 | 0.9045 | 0.9023 | 0.9023 | 0.9102 | 0.9102 | 0.9098 | 0.9098 | 0.9098 | 0.9098 | 0.39% |
| P38 | 0.9148 | 0.9148 | 0.9155 | 0.9163 | 0.9163 | 0.9166 | 0.9174 | 0.9174 | 0.9174 | 0.9174 | 0.11% |
| P39 | 0.9523 | 0.9517 | 0.9518 | 0.9525 | 0.9525 | 0.9521 | 0.9525 | 0.9529 | 0.9521 | 0.9529 | 0.04% |
| P40 | 0.9844 | 0.9844 | 0.9846 | 0.9755 | 0.9766 | 0.9763 | 0.9770 | 0.9770 | 0.9793 | 0.9774 | 0.36% |
| P41 | 0.9857 | 0.9857 | 0.9860 | 0.9868 | 0.9868 | 0.9876 | 0.9876 | 0.9876 | 0.9876 | 0.9876 | 0.08% |
| P42 | 1.0562 | 1.0562 | 1.0558 | 1.0558 | 1.0551 | 1.0539 | 1.0532 | 1.0521 | 1.0521 | 1.0513 | 0.17% |
| P43 | 1.0803 | 1.0809 | 1.0803 | 1.0810 | 1.0810 | 1.0810 | 1.0806 | 1.0810 | 1.0810 | 1.0810 | 0.03% |
| P44 | 1.0879 | 1.0875 | 1.0875 | 1.0868 | 1.0860 | 1.0853 | 1.0849 | 1.0834 | 1.0830 | 1.0830 | 0.17% |
| P45 | 1.1018 | 1.1026 | 1.1026 | 1.1034 | 1.1041 | 1.1041 | 1.1045 | 1.1041 | 1.1041 | 1.1041 | 0.08% |
| P46 | 1.1227 | 1.1272 | 1.1266 | 1.1169 | 1.1169 | 1.1177 | 1.1177 | 1.1177 | 1.1177 | 1.1169 | 0.35% |
| P47 | 1.1535 | 1.1528 | 1.1528 | 1.1524 | 1.1516 | 1.1509 | 1.1505 | 1.1490 | 1.1490 | 1.1490 | 0.14% |
| P48 | 1.1816 | 1.1817 | 1.1816 | 1.1816 | 1.1816 | 1.1816 | 1.1824 | 1.1824 | 1.1824 | 1.1824 | 0.03% |
| P49 | 1.2352 | 1.2351 | 1.2351 | 1.2351 | 1.2351 | 1.2351 | 1.2351 | 1.2359 | 1.2359 | 1.2359 | 0.03% |
| P50 | 1.2777 | 1.2771 | 1.2778 | 1.2840 | 1.2840 | 1.2840 | 1.2848 | 1.2840 | 1.2840 | 1.2840 | 0.24% |
| P51 | 1.2825 | 1.2833 | 1.2833 | 1.3010 | 1.2995 | 1.3010 | 1.2995 | 1.3010 | 1.3010 | 1.3010 | 0.62% |
| P52 | 1.3353 | 1.3338 | 1.3353 | 1.3353 | 1.3372 | 1.3361 | 1.3380 | 1.3372 | 1.3391 | 1.3387 | 0.12% |
| P53 | 1.3146 | 1.3150 | 1.3150 | 1.3150 | 1.3157 | 1.3150 | 1.3165 | 1.3157 | 1.3157 | 1.3157 | 0.04% |
| P54 | 1.3628 | 1.3636 | 1.3636 | 1.3636 | 1.3636 | 1.3632 | 1.3636 | 1.3636 | 1.3636 | 1.3636 | 0.02% |
| P55 | 1.3983 | 1.3976 | 1.3983 | 1.3983 | 1.3983 | 1.3983 | 1.3979 | 1.3983 | 1.3983 | 1.3983 | 0.02% |
| P56 | 1.4368 | 1.4374 | 1.4375 | 1.4375 | 1.4375 | 1.4375 | 1.4375 | 1.4375 | 1.4375 | 1.4375 | 0.01% |
| P57 | 1.4628 | 1.4628 | 1.4628 | 1.4628 | 1.4636 | 1.4644 | 1.4644 | 1.4636 | 1.4636 | 1.4644 | 0.04% |
| P58 | 1.4676 | 1.4676 | 1.4676 | 1.4676 | 1.4676 | 1.4676 | 1.4676 | 1.4676 | 1.4676 | 1.4676 | 0.00% |
| P59 | 1.4981 | 1.4987 | 1.4981 | 1.4981 | 1.4981 | 1.4981 | 1.4981 | 1.4981 | 1.4981 | 1.4981 | 0.01% |
| P60 | 1.5009 | 1.5009 | 1.5009 | 1.5017 | 1.5021 | 1.5021 | 1.5021 | 1.5021 | 1.5021 | 1.5021 | 0.03% |
| P61 | 1.5217 | 1.5164 | 1.5190 | 1.5075 | 1.5075 | 1.5075 | 1.5075 | 1.5079 | 1.5079 | 1.5079 | 0.35% |
| P62 | 1.5304 | 1.5311 | 1.5311 | 1.5311 | 1.5319 | 1.5319 | 1.5319 | 1.5319 | 1.5319 | 1.5319 | 0.03% |
| P63 | 1.5404 | 1.5372 | 1.5365 | 1.5192 | 1.5185 | 1.5181 | 1.5192 | 1.5192 | 1.5192 | 1.5192 | 0.58% |
| P64 | 1.5358 | 1.5358 | 1.5358 | 1.5358 | 1.5358 | 1.5358 | 1.5358 | 1.5365 | 1.5365 | 1.5365 | 0.02% |
| P65 | 1.5621 | 1.5624 | 1.5628 | 1.5632 | 1.5632 | 1.5639 | 1.5639 | 1.5632 | 1.5632 | 1.5632 | 0.04% |
| P66 | 1.6786 | 1.6786 | 1.6786 | 1.6786 | 1.6786 | 1.6786 | 1.6786 | 1.6786 | 1.6786 | 1.6786 | 0.00% |
| P67 | 1.6790 | 1.6797 | 1.6790 | 1.6797 | 1.6805 | 1.6805 | 1.6805 | 1.6797 | 1.6797 | 1.6805 | 0.03% |
| P68 | 1.8146 | 1.8146 | 1.8139 | 1.8067 | 1.8067 | 1.8067 | 1.8067 | 1.8067 | 1.8067 | 1.8067 | 0.19% |
| P69 | 1.8171 | 1.8171 | 1.8165 | 1.8146 | 1.8146 | 1.8139 | 1.8146 | 1.8146 | 1.8127 | 1.8146 | 0.08% |
| P70 | 1.8255 | 1.8255 | 1.8255 | 1.8263 | 1.8263 | 1.8263 | 1.8263 | 1.8263 | 1.8263 | 1.8263 | 0.02% |

**Table S6**

Relative peak areas of common peaks in ten batches of QYSLD

| ESI^+^  Peaks | Samples |  |  |  |  |  |  |  |  |  |  |
| --- | --- | --- | --- | --- | --- | --- | --- | --- | --- | --- | --- |
|  | S1 | S2 | S3 | S4 | S5 | S6 | S7 | S8 | S9 | S10 | CV% |
| P1 | 1.1033 | 1.3402 | 1.7332 | 1.2901 | 0.9561 | 1.3838 | 1.3838 | 1.3151 | 0.9588 | 1.3746 | 17.06% |
| P2 | 0.5754 | 1.1786 | 1.2785 | 1.2312 | 0.7274 | 1.3654 | 1.0377 | 1.5037 | 0.8868 | 1.5893 | 27.49% |
| P3 | 0.2152 | 0.2804 | 0.3098 | 0.3662 | 0.2153 | 0.3072 | 0.2685 | 0.3953 | 0.2010 | 0.3814 | 22.92% |
| P4 | 0.1329 | 0.2098 | 0.1838 | 0.1970 | 0.1150 | 0.2172 | 0.1837 | 0.2119 | 0.1382 | 0.2101 | 19.82% |
| P5 | 0.0574 | 0.0702 | 0.0777 | 0.0778 | 0.0511 | 0.1032 | 0.0819 | 0.0906 | 0.0595 | 0.1079 | 23.20% |
| P6 | 0.6496 | 0.7586 | 0.8209 | 0.0629 | 1.2146 | 2.5264 | 2.1954 | 2.4842 | 0.9859 | 2.4190 | 61.00% |
| P7 | 0.2071 | 0.3833 | 0.3638 | 0.3552 | 0.2109 | 0.3442 | 0.3177 | 0.3322 | 0.2196 | 0.4102 | 22.59% |
| P8 | 2.4096 | 2.5656 | 3.3627 | 3.9970 | 1.9800 | 3.9093 | 3.0677 | 4.1690 | 2.4924 | 4.2812 | 24.63% |
| P9 | 0.0964 | 0.2688 | 0.3083 | 0.2740 | 0.1350 | 0.1933 | 0.3042 | 0.2041 | 0.1238 | 0.3785 | 38.45% |
| P10 | 1.2626 | 2.8474 | 2.7220 | 1.6571 | 1.0151 | 2.3817 | 2.8030 | 3.6203 | 1.0200 | 3.3346 | 40.24% |
| P11 | 0.1730 | 0.5036 | 0.4882 | 1.7493 | 0.1803 | 1.8352 | 1.5424 | 2.2770 | 0.8364 | 2.6177 | 69.56% |
| P12 | 0.1412 | 0.3738 | 0.4321 | 0.3694 | 0.1910 | 0.2921 | 0.3873 | 0.2868 | 0.1736 | 0.4948 | 35.52% |
| P13 | 0.1011 | 0.2826 | 0.2069 | 0.3446 | 0.1164 | 0.4177 | 0.2748 | 0.5316 | 0.2797 | 0.3721 | 42.86% |
| P14 | 0.1359 | 0.2616 | 0.2462 | 0.2898 | 0.1299 | 0.2939 | 0.2488 | 0.3179 | 0.1535 | 0.3295 | 29.52% |
| P15 | 0.0011 | 0.0414 | 0.0292 | 0.0291 | 0.0026 | 0.0238 | 0.0129 | 0.0282 | 0.0367 | 0.0539 | 61.09% |
| P16 | 0.2449 | 0.4436 | 0.3845 | 0.4416 | 0.2406 | 0.4621 | 0.3995 | 0.4982 | 0.2638 | 0.5116 | 25.27% |
| P17 | 0.0058 | 0.1686 | 0.1376 | 0.1910 | 0.0422 | 0.1544 | 0.1102 | 0.1980 | 0.0367 | 0.2261 | 56.67% |
| P18 | 0.0628 | 0.0183 | 0.0173 | 0.0332 | 0.0022 | 0.0152 | 0.0147 | 0.0291 | 0.0035 | 0.0331 | 73.26% |
| P19 | 0.0116 | 0.0186 | 0.0133 | 0.0229 | 0.0109 | 0.0254 | 0.0024 | 0.0048 | 0.0166 | 0.0251 | 50.28% |
| P20 | 0.0102 | 0.0432 | 0.0290 | 0.0598 | 0.0122 | 0.0630 | 0.0450 | 0.0162 | 0.0409 | 0.0642 | 51.19% |
| P21 | 0.0779 | 0.1286 | 0.0869 | 0.1394 | 0.0618 | 0.1579 | 0.1208 | 0.2167 | 0.0877 | 0.1360 | 35.71% |
| P22 | 0.0675 | 0.2475 | 0.3913 | 0.2764 | 0.1057 | 0.3773 | 0.3437 | 0.5401 | 0.0877 | 0.7497 | 63.75% |
| P23 | 0.0464 | 0.0795 | 0.1073 | 0.0935 | 0.0429 | 0.0757 | 0.0889 | 0.0822 | 0.0443 | 0.1217 | 32.65% |
| P24 | 0.2893 | 0.4706 | 0.5359 | 0.6309 | 0.2657 | 0.6151 | 0.5071 | 0.7728 | 0.3675 | 0.7267 | 31.82% |
| P25 | 0.6394 | 1.2676 | 1.1776 | 1.5141 | 0.5958 | 1.4418 | 1.0714 | 1.7794 | 0.5369 | 1.7468 | 37.30% |
| P26 | 0.0058 | 0.0099 | 0.0169 | 0.0135 | 0.0069 | 0.0161 | 0.0176 | 0.0220 | 0.0102 | 0.0242 | 40.77% |
| P27 | 0.0178 | 0.0283 | 0.0285 | 0.0383 | 0.0170 | 0.0481 | 0.0284 | 0.0646 | 0.0209 | 0.0450 | 42.93% |
| P28 | 0.0683 | 0.2842 | 0.3992 | 0.3995 | 0.1387 | 0.2640 | 0.3512 | 0.2565 | 0.1012 | 0.4596 | 46.87% |
| P29 | 0.2050 | 0.3048 | 0.2745 | 0.4348 | 0.1585 | 0.3863 | 0.3467 | 0.4505 | 0.2494 | 0.4183 | 29.62% |
| P30 | 0.1290 | 0.0920 | 0.1008 | 0.1721 | 0.1044 | 0.2338 | 0.1765 | 0.6175 | 0.2723 | 0.1682 | 71.49% |
| P31 | 0.1632 | 0.3111 | 0.3384 | 0.4031 | 0.1778 | 0.4207 | 0.3192 | 0.4790 | 0.1919 | 0.4217 | 33.13% |
| P32 | 0.0482 | 0.0339 | 0.0558 | 0.0606 | 0.0312 | 0.0224 | 0.0877 | 0.0232 | 0.0362 | 0.0474 | 42.36% |
| P33 | 0.3417 | 0.4994 | 0.5475 | 0.6590 | 0.2887 | 0.7293 | 0.4892 | 0.8408 | 0.3774 | 0.7244 | 31.86% |
| P34 | 0.0691 | 0.1385 | 0.1251 | 0.1459 | 0.0695 | 0.1455 | 0.1270 | 0.1772 | 0.1103 | 0.1782 | 27.90% |
| P35 | 0.1112 | 0.1728 | 0.2057 | 0.2450 | 0.2745 | 1.0709 | 0.1508 | 0.3467 | 0.0888 | 3.1046 | 153.29% |
| P36 | 1.4742 | 1.2325 | 0.5906 | 1.6475 | 0.7485 | 2.1698 | 0.9779 | 2.5572 | 1.2817 | 1.3375 | 41.00% |
| P37 | 0.4289 | 0.6149 | 0.7264 | 0.6814 | 0.0682 | 0.5521 | 1.5607 | 1.0390 | 0.7305 | 3.1046 | 84.89% |
| P38 | 0.5410 | 0.5521 | 0.6915 | 0.7103 | 0.2323 | 0.6136 | 0.4785 | 1.1445 | 0.2656 | 1.1869 | 47.06% |
| P39 | 0.2769 | 0.1625 | 0.0355 | 0.2911 | 0.0635 | 0.5357 | 0.0863 | 0.7510 | 0.1922 | 0.1235 | 86.06% |
| P40 | 0.0213 | 0.1014 | 0.1885 | 0.2079 | 0.0564 | 0.1013 | 0.1783 | 0.0958 | 0.0450 | 0.2339 | 57.15% |
| P41 | 0.1074 | 0.3979 | 0.6281 | 0.6706 | 0.1862 | 0.5389 | 0.4870 | 0.5028 | 0.1386 | 0.8899 | 52.61% |
| P42 | 0.3209 | 0.7097 | 0.6768 | 0.6725 | 0.2084 | 0.8222 | 0.6589 | 1.1510 | 0.4763 | 1.0926 | 41.83% |
| P43 | 0.0898 | 0.1664 | 0.2203 | 0.2391 | 0.1074 | 0.2315 | 0.1737 | 0.3390 | 0.1096 | 0.2612 | 38.73% |
| P44 | 1.1032 | 2.1818 | 2.3702 | 2.2855 | 0.7414 | 1.9556 | 1.6132 | 2.5056 | 1.0620 | 2.6254 | 34.62% |
| P45 | 0.2642 | 0.5443 | 1.4289 | 0.8796 | 0.2532 | 0.9752 | 0.6289 | 1.2279 | 0.2859 | 1.0986 | 53.26% |
| P46 | 0.0928 | 0.1853 | 0.0656 | 0.3081 | 0.0601 | 0.4521 | 0.1103 | 0.2579 | 0.0906 | 0.1798 | 66.68% |
| P47 | 0.7513 | 2.0557 | 2.8343 | 1.7047 | 0.7978 | 2.7683 | 1.9788 | 3.6278 | 1.8880 | 3.2373 | 42.10% |
| P48 | 0.2015 | 0.1254 | 0.1442 | 0.2931 | 0.1265 | 0.2231 | 0.1247 | 0.3752 | 0.0972 | 0.2194 | 43.38% |
| P49 | 0.1640 | 0.2846 | 0.3405 | 0.4102 | 0.1629 | 0.3880 | 0.3080 | 0.0670 | 0.2310 | 0.3938 | 39.95% |
| P50 | 0.0071 | 0.0190 | 0.0267 | 0.0363 | 0.0102 | 0.0325 | 0.0148 | 0.0491 | 0.0112 | 0.0409 | 55.45% |
| P51 | 0.5307 | 1.0862 | 1.4144 | 1.6903 | 0.4492 | 1.3528 | 1.0821 | 1.8428 | 0.4543 | 1.7860 | 44.05% |
| P52 | 1.1916 | 3.6779 | 2.3908 | 3.9499 | 1.5044 | 5.1260 | 1.6338 | 2.6669 | 1.4552 | 3.0305 | 45.98% |
| P53 | 0.0718 | 0.1603 | 0.6458 | 0.3567 | 0.1071 | 0.4445 | 0.2846 | 0.5737 | 0.0995 | 0.5181 | 61.96% |
| P54 | 0.3327 | 0.4969 | 0.6464 | 0.8333 | 0.2562 | 0.8382 | 0.5504 | 0.8605 | 0.4024 | 0.6440 | 35.09% |
| P55 | 0.0545 | 0.1240 | 0.1997 | 0.2358 | 0.0565 | 0.2065 | 0.1549 | 0.2198 | 0.0832 | 0.0964 | 45.99% |
| P56 | 0.0265 | 0.0309 | 0.1043 | 0.0671 | 0.0262 | 0.0927 | 0.0518 | 0.1696 | 0.0423 | 0.0828 | 61.46% |
| P57 | 3.1963 | 4.6034 | 5.9029 | 6.8049 | 2.8851 | 4.9725 | 4.8970 | 6.2012 | 2.9907 | 6.4902 | 28.57% |
| P58 | 0.7790 | 1.0624 | 1.1123 | 1.7328 | 0.6805 | 1.9556 | 1.1382 | 2.4504 | 0.9817 | 1.4216 | 39.88% |
| P59 | 0.3235 | 0.6248 | 0.9070 | 1.0933 | 0.3654 | 0.9872 | 0.8430 | 1.1833 | 0.3710 | 0.9007 | 39.61% |
| P60 | 0.9443 | 1.5505 | 2.4664 | 2.2559 | 0.9172 | 2.0940 | 1.6717 | 3.0610 | 1.0115 | 2.9863 | 40.36% |
| P61 | 0.1362 | 0.1563 | 0.9685 | 0.5020 | 0.2730 | 0.3802 | 0.5322 | 0.3689 | 0.3385 | 0.6460 | 54.65% |
| P62 | 0.2881 | 0.4100 | 0.6655 | 0.6640 | 0.3241 | 0.2637 | 0.4817 | 0.4962 | 0.3273 | 0.9220 | 41.29% |
| P63 | 0.4637 | 3.2032 | 4.5183 | 3.0037 | 1.4016 | 1.5641 | 3.2602 | 1.7854 | 2.2098 | 3.0042 | 45.69% |
| P64 | 0.1573 | 0.5790 | 1.0293 | 0.5979 | 0.2511 | 0.4843 | 0.5581 | 0.6332 | 0.3322 | 0.6625 | 44.10% |
| P65 | 1.3951 | 1.0874 | 2.9590 | 2.5714 | 2.1400 | 0.4858 | 2.1371 | 1.5972 | 1.7956 | 3.7149 | 44.76% |
| P66 | 0.2422 | 0.4952 | 0.8227 | 0.5674 | 0.2628 | 0.6170 | 0.4615 | 1.0117 | 0.2584 | 0.6014 | 44.53% |
| P67 | 0.1457 | 0.0540 | 0.5779 | 0.3863 | 0.8441 | 0.4227 | 0.2837 | 0.7567 | 0.1164 | 0.5543 | 61.88% |
| P68 | 0.5522 | 0.4302 | 0.4469 | 0.4686 | 0.4686 | 0.4456 | 0.4619 | 0.4451 | 0.5849 | 0.4977 | 10.01% |
| P69 | 0.9670 | 1.5240 | 1.6555 | 1.4356 | 1.4356 | 0.9890 | 1.1868 | 1.0919 | 1.3018 | 1.2964 | 16.93% |
| P70 | 1.1618 | 1.2527 | 1.2902 | 1.0395 | 1.0395 | 0.9740 | 1.0282 | 1.0023 | 1.0436 | 1.0707 | 9.37% |

**Table S7**

MS data and identification results of common peaks of QYSLD

| No | tR  /min | Formula | Observed（m/z） | Calculated（m/z） | Adduct | Error  (ppm) | Fragment ions (m/z) | Identification | Source |
| --- | --- | --- | --- | --- | --- | --- | --- | --- | --- |
| P1 | 1.09 | C_6_H_14_N_4_O_2_ | 175.1193 | 175.1195 | [M+H]^+^ | -1.1 | 131.0082, 113.9642 | L-Arginine | g,h |
| P2 | 1.66 | C_5_H_4_N_4_O | 137.0452 | 137.0458 | [M+H]^+^ | -4.3 | 119.0340, 110.0582 | Hypoxanthine | g,h |
| P3 | 1.90 | C_9_H_11_NO_3_ | 182.0801 | 182.0812 | [M+H]^+^ | -6.0 | 165.0534, 138.0505 | Tyrosine | h |
| P4 | 2.22 | C_16_H_22_O_11_ | 389.1073 | 389.1084 | [M−H]^−^ | -2.8 | 227.0563, 209.0443, 165.0549 | Deacetyl asperulosidic acid | c |
| P5 | 2.49 | C_10_H_12_N_4_O_5_ | 267.0724 | 267.0735 | [M−H]^−^ | -4.1 | 150.0408, 135.0306 | Inosine | h |
| P6 | 2.53 | C_5_H_5_N_5_O | 152.0569 | 152.0564 | [M+H]^+^ | 3.2 | 135.0288, 110.0346 | Guanine | h |
| P7 | 3.55 | C_16_H_22_O_11_ | 389.1073 | 389.1084 | [M−H]^−^ | -2.8 | 227.0563, 209.0443, 165.0549, 147.0430 | Monotropein | c |
| P8 | 3.67 | C_9_H_11_NO_2_ | 166.0853 | 166.0868 | [M+H]^+^ | -9.0 | 120.0787, 89.0305 | L-Phenylalanine | h |
| P9 | 6.06 | C_17_H_24_O_11_ | 449.1322 | 449.1295 | [M+HCOO]^−^ | 6.0 | 373.1165, 241.0679, 223.0531, 191.0236 | Deacetyl asperulosidic acid methyl ester | c |
| P10 | 6.36 | C_9_H_7_NO_2_ | 162.0550 | 162.0555 | [M+H]^+^ | -3.0 | 146.0599, 144.0468, 130.0639 | 3,8-diol-Quinoline | g |
| P11 | 6.57 | C_9_H_11_NO_2_ | 188.0695 | 188.0682 | [M+Na]^+^ | 6.9 | 115.0545, 89.0402 | D-*β*-phenylalanine | h |
| P12 | 7.57 | C_17_H_24_O_11_ | 449.1308 | 449.1295 | [M+HCOO]^−^ | 2.9 | 241.0711 | Scandoside methyl ester | c |
| P13 | 7.94 | C_9_H_14_O_6_ | 217.0700 | 217.0718 | [M−H]^−^ | -8.2 | 161.0388, 115.0714 | Triacetin | d |
| P14 | 8.67 | C_17_H_24_O_12_ | 419.1199 | 419.1195 | [M−H]^−^ | 0.9 | 401.1065, 257.0748, 239.0483, 221.0406 | 7-Formylipolamiidic acid | d |
| P15 | 8.70 | C_11_H_10_O_5_ | 223.0612 | 223.0601 | [M+H]^+^ | 4.9 | 207.0664, 205.0752, 191.0355 | Isofraxidin | i |
| P16 | 8.9 | C_18_H_24_O_12_ | 431.1193 | 431.1195 | [M−H]^−^ | 0.4 | 251.0537, 165.0549, 135.0425 | Asperulosidic acid | c |
| P17 | 8.94 | C_10_H_6_O_3_ | 175.0408 | 175.0395 | [M+H]^+^ | 7.4 | 131.0527, 115.0567 | 6-Formaldehydecoumarin | e |
| P18 | 9.23 | - | 217.0974 | - | [M+H]^+^ | - | 144.0812, 115.0545 | Unknown | - |
| P19 | 9.55 | C_16_H_18_O_9_ | 353.0873 | 353.0878 | [M−H]^−^ | -1.4 | 191.0575 | Chlorogenic acid | c |
| P20 | 10.5 | C_9_H_8_O_4_ | 179.0336 | 179.0350 | [M−H]^−^ | -7.8 | 161.0439, 135.0425 | Caffeic acid | a |
| P21 | 10.9 | - | 459.114 | - | [M−H]^−^ | - | - | Unknown | - |
| P22 | 12.13 | - | 192.0661 | - | [M+H]^+^ | - | 177.0414, 149.0471, 120.0451 | Unknown | - |
| P23 | 14.44 | C_9_H_8_O_3_ | 163.0393 | 163.0395 | [M−H]^−^ | -1.2 | 119.0499, 93.0303 | *p*-Coumaric acid | c |
| P24 | 15.30 | C_27_H_30_O_17_ | 625.143 | 625.1410 | [M−H]^−^ | 3.2 | 445.0778, 300.0269 | Heliosin | i |
| P25 | 15.8 | C_15_H_22_O_2_ | 235.1692 | 235.1698 | [M+H]^+^ | -2.5 | 217.1577 | Neoprocurcumenol | e |
| P26 | 16.13 | C_9_H_8_O_3_ | 163.0393 | 163.0395 | [M−H]^−^ | -1.2 | 119.0499 | *o*-Coumaric Acid | c |
| P27 | 16.89 | C_26_H_28_O_16_ | 595.1304 | 595.1305 | [M−H]^−^ | -0.1 | 300.0284 | Quercetin-3-sambubioside | c |
| P28 | 18.44 | C_22_H_22_O_10_ | 491.1179 | 491.1190 | [M+HCOO]^−^ | -2.2 | 283.0594, 268.0364, 211.0403, 135.0068 | Calycosin7-*O*-*β*-D-Glucopyranoside | a |
| P29 | 19.07 | C_27_H_30_O_16_ | 609.1451 | 609.1456 | [M−H]^−^ | -0.8 | 300.0284 | Rutin | a,c,i |
| P30 | 20.34 | C_28_H_24_O_16_ | 615.0991 | 615.0992 | [M−H]^−^ | -0.1 | 301.0324 | 2'-*O*-Galloylhyperin | c |
| P31 | 21.51 | C_38_H_40_O_21_ | 831.2023 | 831.1984 | [M−H]^−^ | 4.6 | 625.1430, 300.0284 | Quercetin-3-*O*-[2-*O*-(6-*O*-*E*-sinapoyl)-*β*-D-glueopyranosyl]-*β*-D-galactopyranoside | c |
| P32 | 21.85 | C_27_H_41_NO_3_ | 428.3169 | 428.3165 | [M+H]^+^ | 0.9 | 410.3078, 393.2817, 114.0909 | Peimisine | j |
| P33 | 22.27 | C_37_H_38_O_20_ | 801.1888 | 801.1878 | [M−H]^−^ | 1.2 | 625.1430, 300.0284 | Quercetin-3-*O*-[2-*O*-(6-*O*-*E*-feruloyl)-*β*-D-glueopyranosyl]-*β*-D-glucopyranosyl | c |
| P34 | 22.82 | C_9_H_16_O_4_ | 187.0957 | 187.0970 | [M−H]^−^ | -6.9 | 169.0140, 125.0975 | Azelaic acid | a |
| P35 | 23.31 | - | 685.3624 | - | [M+H]^+^ | - | 540.3198, 358.2101, 342.1317 | Unknown | - |
| P36 | 23.73 | C_26_H_30_O_13_ | 549.1627 | 549.1608 | [M−H]^−^ | 3.4 | 595.1710, 369.0956 | *Z*-6-*O*-*p*-Coumaroyl scandoside methyl ester | c |
| P37 | 23.75 | - | 628.3414 | - | [M+H]^+^ | - | - | Unknown | - |
| P38 | 24.02 | C_15_H_20_O_2_ | 233.1523 | 233.1542 | [M+H]^+^ | -8.1 | 215.1394, 185.1044 | Curcumafuranol | e |
| P39 | 24.98 | C_26_H_30_O_13_ | 549.1627 | 549.1608 | [M−H]^−^ | 3.4 | 387.1037, 369.0995 | *E*-6-*O*-*p*-Coumaroyl scandoside methyl ester | c |
| P40 | 25.88 | - | 475.1239 | - | [M−H]^−^ | - | - | Unknown | - |
| P41 | 25.89 | - | 431.1287 | - | [M+H]^+^ | - | 269.0802, 254.0596 | Unknown | - |
| P42 | 27.85 | C_45_H_73_NO_16_ | 884.5004 | 884.5008 | [M+H]^+^ | -0.4 | 738.4427, 722.4520, 576.3888, 414.3333, 396.3305, 271.2087, 253.1962 | Solasonine | b |
| P43 | 28.38 | C_21_H_28_O_6_ | 375.1788 | 375.1813 | [M−H]^−^ | -6.6 | 360.1552, 151.0401 | Octahydrocurcumin | e |
| P44 | 28.65 | C_45_H_73_NO_15_ | 868.5041 | 868.5053 | [M+H]^+^ | -1.3 | 722.4520, 576.3937, 414.3375 | Solamargine | b |
| P45 | 28.95 | C_16_H_12_O_5_ | 285.0776 | 285.0757 | [M+H]^+^ | 6.6 | 270.0543, 213.0559, 137.0236 | Calycosin | a |
| P46 | 29.65 | C_27_H_43_NO_2_ | 414.3375 | 414.3372 | [M+H]^+^ | 0.7 | 271.2020, 253.1897 | Solasodine | b |
| P47 | 30.31 | C_39_H_63_NO_11_ | 722.4465 | 722.4479 | [M+H]^+^ | -1.9 | 576.3888, 414.3333, 271.2053, 253.1962 | Solasurine | b |
| P48 | 31.18 | C_39_H_62_O_15_ | 815.4135 | 815.4144 | [M+HCOO]^−^ | -1.1 | 769.4039, 623.3484 | Soladulcoside A | a |
| P49 | 32.58 | C_18_H_32_O_4_ | 327.2164 | 327.2177 | [M−H]^−^ | -3.9 | 229.1455, 211.1325, 171.1008 | 9(*S*),12(*S*),13(*S*)-Trihydroxy-10(*E*),15(*Z*)-octadecadienoic acid | d |
| P50 | 33.65 | C_15_H_18_O_3_ | 247.1355 | 247.1334 | [M+H]^+^ | 8.4 | 229.1275, 199.0698 | Zedoarol | e |
| P51 | 33.89 | C_15_H_22_O_2_ | 235.1716 | 235.1698 | [M+H]^+^ | 7.6 | 217.1577, 203.1023, 199.1477 | Isocurcumenol | e |
| P52 | 34.45 | C_15_H_16_O_4_ | 259.0966 | 259.0970 | [M−H]^−^ | -1.5 | 244.0766, 188.0121, 172.9904 | Linderane | e |
| P53 | 34.65 | C_16_H_12_O_4_ | 269.0802 | 269.0814 | [M+H]^+^ | -4.4 | 253.0497, 237.0578 | Formononetin | a |
| P54 | 36.00 | C_43_H_70_O_15_ | 871.4727 | 871.4691 | [M+HCOO]^−^ | 4.1 | 825.4482, 765.4413, 625.3426 | Astragaloside II | a |
| P55 | 36.89 | C_43_H_70_O_15_ | 871.4727 | 871.4691 | [M+HCOO]^−^ | 4.1 | 825.4718, 765.4583, 625.3630 | Isoastragaloside II | a |
| P56 | 37.90 | C_15_H_10_O_3_ | 237.0560 | 237.0552 | [M−H]^−^ | 3.3 | 209.0621, 195.0466 | 2-Hydroxy-3-methylanthraquinone | c |
| P57 | 38.66 | C_15_H_22_O_2_ | 235.1716 | 235.1698 | [M+H]^+^ | 7.6 | 257.1539, 217.1607, 199.1506, 159.1176 | 13-Hydroxygermacrone | e |
| P58 | 38.73 | C_45_H_72_O_16_ | 913.4871 | 913.4797 | [M+HCOO]^−^ | 8.1 | 867.4841、825.4718、807.3578 | Astragaloside I | a |
| P59 | 39.54 | C_45_H_72_O_16_ | 913.4871 | 913.4797 | [M+HCOO]^−^ | 8.1 | 867.4720、825.4542、807.4741 | Isoastragaloside I | a |
| P60 | 39.66 | C_15_H_18_O_3_ | 247.1323 | 247.1334 | [M+H]^+^ | -4.4 | 269.1171, 211.1163, 196.0905, 181.0656 | Curcolone | e |
| P61 | 40.03 | - | 1063.5593 | - | [M−H]^−^ | - | 1109.5537, 1031.5238 | Unknown | - |
| P62 | 40.46 | C_15_H_24_O_2_ | 237.1870 | 237.1849 | [M+H]^+^ | 8.8 | 161.1343, 135.1192 | Curcumol | e |
| P63 | 40.47 | - | 1033.5294 | - | [M−H]^−^ | - | 1079.5422 | Unknown | - |
| P64 | 40.81 | - | 1149.5800 | - | [M−H]^−^ | - | 1195.5880 | Unknown | - |
| P65 | 41.29 | C_15_H_24_O_2_ | 237.1838 | 237.1849 | [M+H]^+^ | -4.6 | 219.1763, 207.0575, 135.1192 | Curdione | e |
| P66 | 44.39 | C_18_H_32_O_3_ | 295.2272 | 295.2279 | [M−H]^−^ | -2.3 | 277.2198, 171.1035 | 13-Hydroxylinoleic acid | d |
| P67 | 44.43 | C_18_H_30_O_2_ | 279.2321 | 279.2319 | [M+H]^+^ | 0.7 | 264.2792, 229.1491 | Linolenic acid | d |
| P68 | 48.09 | C_30_H_48_O_3_ | 455.3508 | 455.3525 | [M−H]^−^ | -3.7 | 501.3563, 339.2293 | Ursolic acid | c |
| P69 | 48.19 | C_20_H_32_O_2_ | 303.2306 | 303.2324 | [M−H]^−^ | -5.9 | 259.2449, 217.2117 | Arachidonic acid | h |
| P70 | 48.39 | C_18_H_32_O_2_ | 279.2302 | 279.2324 | [M−H]^−^ | -7.8 | 200.8566, 199.8486 | Linoleic acid | d |

a: *Astragalus mongholicus* Bunge; b: *Solanum nigrum* L., c: *Hedyotis diffusa* Willd., d: [*Coix lacryma-jobi* L.](https://mpns.science.kew.org/mpns-portal/plantDetail?plantId=405633&query=%E8%96%8F%E8%8B%A1%E4%BB%81&filter=&fuzzy=false&nameType=all&dbs=wcs), e: *Curcuma phaeocaulis* Valeton, f: [*Polygonatum odoratum* (Mill.) Druce](https://mpns.science.kew.org/mpns-portal/plantDetail?plantId=284008&query=%E7%8E%89%E7%AB%B9&filter=&fuzzy=false&nameType=all&dbs=wcs), g: *Scolopendra subspinipes mutilans* L.Koch, h: *Pheretima aspergillum* (E. Perrier), i: *Euphorbia helioscopia* L., j: *Fritillaria cirrhosa* D.Don

**Table S8**

Correlations and grade of GRA between common peaks and the antioxidant, anti-NSCLC activities, respectively

| Peaks | DPPH |  | | FRAP |  | CCK-8 |  | Woung healing |  | Transwell migration |  | Transwell invasion |  |
| --- | --- | --- | --- | --- | --- | --- | --- | --- | --- | --- | --- | --- | --- |
|  | Correlations | | Rank | Correlations | Rank | Correlations | Rank | Correlations | Rank | Correlations | Rank | Correlations | Rank |
| P1 | 0.8736 | 6 | | 0.8279 | 5 | 0.8280 | 4 | 0.8984 | 7 | 0.7929 | 37 | 0.8657 | 7 |
| P2 | 0.8568 | 15 | | 0.8231 | 8 | 0.8045 | 10 | 0.9059 | 4 | 0.7803 | 50 | 0.8485 | 19 |
| P4 | 0.8772 | 4 | | 0.8140 | 16 | 0.8072 | 9 | 0.9093 | 3 | 0.7948 | 34 | 0.8684 | 6 |
| P5 | 0.8506 | 22 | | 0.8285 | 4 | 0.7890 | 23 | 0.8921 | 9 | 0.7798 | 51 | 0.8417 | 28 |
| P6 | 0.8156 | 51 | | 0.7853 | 38 | 0.7778 | 38 | 0.8399 | 41 | 0.7869 | 44 | 0.8054 | 50 |
| P7 | 0.8698 | 8 | | 0.8248 | 7 | 0.7891 | 21 | 0.8844 | 12 | 0.7914 | 39 | 0.8651 | 8 |
| P8 | 0.8496 | 24 | | 0.8124 | 18 | 0.7796 | 37 | 0.8912 | 10 | 0.8036 | 20 | 0.8420 | 25 |
| P9 | 0.8087 | 52 | | 0.7830 | 39 | 0.7821 | 33 | 0.8154 | 50 | 0.7927 | 38 | 0.8055 | 49 |
| P10 | 0.8688 | 9 | | 0.8171 | 14 | 0.8148 | 7 | 0.8409 | 40 | 0.7882 | 42 | 0.8419 | 27 |
| P12 | 0.8653 | 11 | | 0.8213 | 10 | 0.7890 | 22 | 0.8599 | 33 | 0.8122 | 10 | 0.8534 | 15 |
| P13 | 0.8535 | 19 | | 0.7821 | 40 | 0.7827 | 31 | 0.8804 | 13 | 0.8147 | 8 | 0.8572 | 12 |
| P14 | 0.8465 | 28 | | 0.8133 | 17 | 0.7678 | 46 | 0.8801 | 14 | 0.8027 | 22 | 0.8308 | 38 |
| P15 | 0.6744 | 59 | | 0.5374 | 59 | 0.6520 | 58 | 0.6404 | 59 | 0.7183 | 57 | 0.6138 | 59 |
| P16 | 0.8564 | 17 | | 0.8120 | 19 | 0.7812 | 35 | 0.8924 | 8 | 0.7994 | 26 | 0.8435 | 24 |
| P17 | 0.8424 | 32 | | 0.8004 | 26 | 0.7569 | 55 | 0.8644 | 29 | 0.7989 | 27 | 0.8325 | 36 |
| P18 | 0.6937 | 58 | | 0.5440 | 58 | 0.6422 | 59 | 0.6663 | 58 | 0.6914 | 59 | 0.6603 | 58 |
| P19 | 0.8382 | 35 | | 0.8110 | 22 | 0.7963 | 14 | 0.8654 | 26 | 0.7954 | 33 | 0.8276 | 41 |
| P20 | 0.8384 | 34 | | 0.7871 | 35 | 0.7669 | 47 | 0.8631 | 31 | 0.8045 | 18 | 0.8420 | 26 |
| P21 | 0.8567 | 16 | | 0.7980 | 30 | 0.7863 | 27 | 0.9058 | 5 | 0.8060 | 15 | 0.8537 | 14 |
| P22 | 0.8288 | 40 | | 0.7751 | 43 | 0.7762 | 39 | 0.8319 | 44 | 0.8105 | 11 | 0.8361 | 33 |
| P23 | 0.8618 | 13 | | 0.8193 | 11 | 0.7862 | 28 | 0.8685 | 20 | 0.8153 | 7 | 0.8629 | 10 |
| P24 | 0.8889 | 1 | | 0.7201 | 54 | 0.7959 | 15 | 0.8603 | 32 | 0.8585 | 1 | 0.9339 | 1 |
| P25 | 0.8435 | 31 | | 0.7993 | 29 | 0.7826 | 32 | 0.8788 | 15 | 0.7994 | 25 | 0.8368 | 32 |
| P26 | 0.8658 | 10 | | 0.8178 | 13 | 0.7733 | 42 | 0.8582 | 35 | 0.8085 | 13 | 0.8484 | 20 |
| P28 | 0.8784 | 3 | | 0.6943 | 56 | 0.8240 | 5 | 0.8339 | 43 | 0.8584 | 2 | 0.9063 | 3 |
| P29 | 0.8491 | 25 | | 0.7929 | 32 | 0.7950 | 17 | 0.9169 | 1 | 0.7980 | 28 | 0.8327 | 35 |
| P30 | 0.8308 | 39 | | 0.7599 | 49 | 0.7668 | 48 | 0.8651 | 27 | 0.7946 | 35 | 0.7685 | 54 |
| P32 | 0.7187 | 57 | | 0.6172 | 57 | 0.7887 | 25 | 0.7006 | 57 | 0.6941 | 58 | 0.7084 | 57 |
| P34 | 0.8721 | 7 | | 0.8117 | 21 | 0.8007 | 12 | 0.9145 | 2 | 0.8015 | 23 | 0.8686 | 5 |
| P35 | 0.8578 | 14 | | 0.8251 | 6 | 0.7820 | 34 | 0.8673 | 24 | 0.7892 | 41 | 0.8450 | 23 |
| P36 | 0.8211 | 48 | | 0.7855 | 37 | 0.7888 | 24 | 0.8680 | 22 | 0.7770 | 53 | 0.7649 | 55 |
| P37 | 0.7888 | 54 | | 0.8402 | 3 | 0.8082 | 8 | 0.8173 | 49 | 0.7657 | 55 | 0.7764 | 53 |
| P38 | 0.7851 | 55 | | 0.7422 | 53 | 0.7430 | 57 | 0.7711 | 56 | 0.7697 | 54 | 0.7885 | 52 |
| P39 | 0.8374 | 36 | | 0.8119 | 20 | 0.7965 | 13 | 0.8006 | 54 | 0.7833 | 48 | 0.8215 | 44 |
| P42 | 0.8476 | 27 | | 0.8026 | 24 | 0.7836 | 30 | 0.8684 | 21 | 0.8089 | 12 | 0.8454 | 22 |
| P43 | 0.8270 | 41 | | 0.7798 | 42 | 0.7643 | 50 | 0.8308 | 45 | 0.8010 | 24 | 0.8301 | 39 |
| P44 | 0.8478 | 26 | | 0.8012 | 25 | 0.8721 | 2 | 0.8662 | 25 | 0.8182 | 6 | 0.8501 | 18 |
| P46 | 0.7817 | 56 | | 0.8487 | 1 | 0.7539 | 56 | 0.7981 | 55 | 0.7640 | 56 | 0.7524 | 56 |
| P47 | 0.8503 | 23 | | 0.7863 | 36 | 0.7952 | 16 | 0.8575 | 36 | 0.8246 | 5 | 0.8525 | 16 |
| P48 | 0.8342 | 38 | | 0.8050 | 23 | 0.8234 | 6 | 0.8651 | 28 | 0.7772 | 52 | 0.8063 | 47 |
| P49 | 0.8445 | 29 | | 0.7922 | 34 | 0.7807 | 36 | 0.8679 | 23 | 0.7958 | 31 | 0.8380 | 31 |
| P50 | 0.8196 | 49 | | 0.8182 | 12 | 0.7578 | 54 | 0.8263 | 47 | 0.7974 | 29 | 0.8011 | 51 |
| P51 | 0.8418 | 33 | | 0.7930 | 31 | 0.7709 | 44 | 0.8760 | 16 | 0.7893 | 40 | 0.8350 | 34 |
| P52 | 0.8261 | 43 | | 0.7637 | 47 | 0.7687 | 45 | 0.8288 | 46 | 0.7853 | 46 | 0.8387 | 30 |
| P54 | 0.8231 | 47 | | 0.7819 | 41 | 0.7608 | 53 | 0.8591 | 34 | 0.7858 | 45 | 0.8219 | 43 |
| P55 | 0.8232 | 46 | | 0.7549 | 50 | 0.7609 | 52 | 0.8055 | 53 | 0.7828 | 49 | 0.8234 | 42 |
| P56 | 0.8179 | 50 | | 0.7484 | 52 | 0.7630 | 51 | 0.8374 | 42 | 0.8032 | 21 | 0.8078 | 46 |
| P58 | 0.8269 | 42 | | 0.7665 | 46 | 0.7754 | 41 | 0.8753 | 17 | 0.7880 | 43 | 0.8056 | 48 |
| P59 | 0.8372 | 37 | | 0.7733 | 44 | 0.7760 | 40 | 0.8239 | 48 | 0.8044 | 19 | 0.8568 | 13 |
| P60 | 0.8511 | 20 | | 0.7996 | 28 | 0.7911 | 20 | 0.8573 | 37 | 0.8072 | 14 | 0.8517 | 17 |
| P61 | 0.8543 | 18 | | 0.7924 | 33 | 0.7914 | 19 | 0.8445 | 38 | 0.8261 | 4 | 0.8648 | 9 |
| P62 | 0.8770 | 5 | | 0.7720 | 45 | 0.8038 | 11 | 0.8712 | 18 | 0.8047 | 17 | 0.9101 | 2 |
| P63 | 0.8508 | 21 | | 0.8000 | 27 | 0.7877 | 26 | 0.9044 | 6 | 0.7964 | 30 | 0.8462 | 21 |
| P64 | 0.8238 | 44 | | 0.7510 | 51 | 0.7846 | 29 | 0.8132 | 52 | 0.8269 | 3 | 0.8318 | 37 |
| P65 | 0.8008 | 53 | | 0.7077 | 55 | 0.7659 | 49 | 0.8143 | 51 | 0.7941 | 36 | 0.8404 | 29 |
| P66 | 0.8443 | 30 | | 0.7614 | 48 | 0.7936 | 18 | 0.8436 | 39 | 0.8133 | 9 | 0.8283 | 40 |
| P67 | 0.8234 | 45 | | 0.8218 | 9 | 0.7733 | 43 | 0.8694 | 19 | 0.7956 | 32 | 0.8162 | 45 |
| P68 | 0.8641 | 12 | | 0.8423 | 2 | 0.8798 | 1 | 0.8637 | 30 | 0.7835 | 47 | 0.8599 | 11 |
| P70 | 0.8883 | 2 | | 0.8159 | 15 | 0.8624 | 3 | 0.8897 | 11 | 0.8054 | 16 | 0.8852 | 4 |

**Table S9**

Correlation coefficient and VIP values of PLSR between common peaks and the antioxidant, anti-NSCLC activities, respectively

| Peaks | DPPH |  | FRAP |  | CCK-8 |  | Woung healing |  | Transwell migration |  | Transwell invasion |  |
| --- | --- | --- | --- | --- | --- | --- | --- | --- | --- | --- | --- | --- |
|  | Correlations | VIP | Correlations | VIP | Correlations | VIP | Correlations | VIP | Correlations | VIP | Correlations | VIP |
| P1 | 0.0544 | 1.0462 | 0.0232 | 0.9758 | 0.0782 | 1.0988 | 0.0430 | 1.0370 | 0.0583 | 0.9985 | 0.0388 | 1.0284 |
| P2 | 0.0148 | 0.9798 | 0.0203 | 0.9703 | 0.0382 | 0.9630 | 0.0458 | 1.0399 | -0.0021 | 0.9299 | 0.0207 | 0.9965 |
| P4 | 0.0391 | 1.0161 | 0.1470 | 1.4132 | 0.0478 | 0.9924 | 0.0365 | 1.0295 | 0.0429 | 0.9700 | 0.0425 | 1.0396 |
| P5 | -0.0012 | 0.9793 | 0.0479 | 1.0245 | 0.0284 | 0.9470 | 0.0245 | 1.0128 | -0.0190 | 0.9442 | 0.0411 | 1.0337 |
| P6 | -0.0237 | 0.9747 | 0.0006 | 0.9444 | -0.0318 | 0.9485 | -0.0086 | 0.9826 | -0.0177 | 0.9230 | -0.0126 | 0.9739 |
| P7 | 0.0059 | 0.9795 | 0.0562 | 1.0119 | 0.0197 | 0.9351 | 0.0154 | 1.0051 | 0.0025 | 0.9328 | 0.0362 | 1.0243 |
| P8 | 0.0021 | 0.9780 | 0.0243 | 0.9778 | 0.0090 | 0.9261 | 0.0218 | 1.0094 | 0.0097 | 0.9328 | 0.0179 | 0.9962 |
| P9 | -0.0291 | 0.9757 | 0.0508 | 1.0078 | -0.0288 | 0.9353 | -0.0464 | 1.0197 | -0.0540 | 0.9804 | -0.0013 | 0.9617 |
| P10 | 0.0393 | 0.9819 | 0.0696 | 1.0795 | 0.0483 | 0.9624 | -0.0274 | 0.9820 | 0.0615 | 0.9777 | 0.0470 | 1.0162 |
| P12 | 0.0183 | 0.9800 | 0.0345 | 0.9895 | 0.0168 | 0.9263 | -0.0136 | 0.9986 | 0.0400 | 0.9575 | 0.0371 | 1.0201 |
| P13 | 0.0067 | 0.9671 | 0.0030 | 0.9521 | -0.0155 | 0.9262 | 0.0420 | 1.0243 | 0.0160 | 0.9245 | 0.0184 | 0.9852 |
| P14 | -0.0093 | 0.9805 | 0.0316 | 0.9885 | -0.0040 | 0.9265 | -0.0002 | 0.9993 | -0.0074 | 0.9339 | 0.0180 | 0.9959 |
| P15 | 0.1010 | 0.9416 | -0.0957 | 0.9564 | 0.0368 | 0.6507 | 0.0911 | 0.8697 | 0.1887 | 1.3571 | 0.0221 | 0.6512 |
| P16 | 0.0058 | 0.9817 | 0.0316 | 0.9918 | 0.0162 | 0.9337 | 0.0143 | 1.0070 | 0.0099 | 0.9358 | 0.0254 | 1.0082 |
| P17 | -0.0236 | 0.9788 | 0.0714 | 1.0683 | -0.0125 | 0.9194 | 0.0003 | 0.9831 | -0.0216 | 0.9315 | 0.0303 | 0.9972 |
| P18 | 0.0403 | 0.7761 | -0.1410 | 1.2916 | -0.0781 | 0.9380 | 0.0216 | 0.7562 | -0.0418 | 0.7487 | 0.0143 | 0.7389 |
| P19 | -0.0033 | 0.9270 | 0.0300 | 0.9378 | 0.0143 | 0.8809 | 0.0250 | 0.9606 | 0.0430 | 0.9203 | 0.0171 | 0.9442 |
| P20 | -0.0167 | 0.9707 | 0.0053 | 0.9482 | -0.0415 | 0.9774 | 0.0488 | 1.0330 | -0.0236 | 0.9333 | 0.0124 | 0.9758 |
| P21 | 0.0280 | 0.9994 | -0.0064 | 0.9690 | 0.0264 | 0.9466 | 0.0274 | 1.0195 | 0.0352 | 0.9585 | 0.0284 | 1.0133 |
| P22 | 0.0104 | 0.9624 | -0.0350 | 0.9865 | -0.0269 | 0.9391 | -0.0128 | 0.9869 | -0.0012 | 0.9152 | -0.0032 | 0.9708 |
| P23 | 0.0208 | 0.9904 | 0.0291 | 0.9875 | 0.0106 | 0.9295 | 0.0295 | 1.0075 | 0.0362 | 0.9592 | 0.0291 | 1.0137 |
| P24 | 0.1245 | 1.2725 | -0.0412 | 0.9734 | 0.0618 | 0.9963 | 0.0729 | 1.0624 | 0.2001 | 1.5639 | 0.1150 | 1.2769 |
| P25 | -0.0039 | 0.9865 | 0.0183 | 0.9784 | -0.0015 | 0.9330 | 0.0095 | 1.0099 | -0.0134 | 0.9454 | 0.0017 | 0.9959 |
| P26 | -0.0098 | 0.9685 | 0.0519 | 1.0351 | -0.0001 | 0.9136 | -0.0182 | 0.9934 | 0.0054 | 0.9198 | 0.0346 | 1.0078 |
| P28 | 0.1575 | 1.4233 | -0.0640 | 1.0199 | 0.0950 | 1.1234 | 0.0668 | 1.0198 | 0.2444 | 1.7976 | 0.1371 | 1.3765 |
| P29 | 0.0075 | 0.9787 | 0.0020 | 0.9621 | -0.0010 | 0.9252 | 0.0509 | 1.0523 | -0.0180 | 0.9418 | 0.0052 | 0.9882 |
| P30 | 0.0104 | 0.8903 | -0.0334 | 0.9132 | -0.0081 | 0.8441 | 0.1069 | 1.1425 | -0.0490 | 0.9114 | -0.0149 | 0.9050 |
| P32 | 0.1236 | 1.1197 | 0.0393 | 0.7584 | 0.2428 | 2.0234 | 0.0589 | 0.8227 | 0.0568 | 0.7676 | 0.1079 | 1.0848 |
| P34 | 0.0335 | 1.0085 | 0.0274 | 0.9890 | 0.0300 | 0.9556 | 0.0303 | 1.0259 | 0.0297 | 0.9547 | 0.0304 | 1.0201 |
| P35 | -0.0109 | 0.9849 | 0.0475 | 1.0253 | -0.0025 | 0.9290 | 0.0449 | 1.0317 | -0.0308 | 0.9607 | 0.0138 | 1.0016 |
| P36 | -0.0094 | 0.9342 | -0.0146 | 0.9246 | 0.0146 | 0.8849 | 0.0788 | 1.0800 | -0.0405 | 0.9315 | -0.0278 | 0.9653 |
| P37 | -0.0471 | 0.9907 | 0.0924 | 1.1579 | 0.0648 | 1.0060 | -0.0052 | 0.9512 | -0.0906 | 1.0767 | -0.0335 | 0.9751 |
| P38 | -0.0014 | 0.8846 | 0.0570 | 0.9655 | -0.0029 | 0.8377 | 0.0312 | 0.9256 | 0.0351 | 0.8686 | 0.0209 | 0.9060 |
| P39 | 0.0650 | 1.0045 | 0.0758 | 1.0467 | 0.0311 | 0.9551 | 0.0130 | 0.9228 | 0.0716 | 0.9667 | 0.0204 | 0.9206 |
| P42 | 0.0135 | 0.9857 | 0.0298 | 0.9896 | 0.0088 | 0.9296 | 0.0065 | 1.0050 | 0.0291 | 0.9508 | 0.0091 | 0.9943 |
| P43 | -0.0108 | 0.9719 | -0.0042 | 0.9540 | -0.0332 | 0.9600 | -0.0146 | 0.9955 | -0.0320 | 0.9503 | -0.0247 | 0.9971 |
| P44 | 0.0245 | 0.9987 | 0.0092 | 0.9695 | 0.1726 | 1.5911 | 0.0072 | 1.0065 | 0.0619 | 1.0116 | 0.0141 | 0.9982 |
| P46 | -0.0847 | 1.0885 | 0.0962 | 1.1601 | -0.0361 | 0.9101 | -0.0953 | 1.1129 | -0.0948 | 1.0724 | -0.0069 | 0.9153 |
| P47 | 0.0281 | 0.9912 | -0.0046 | 0.9602 | 0.0045 | 0.9204 | 0.0136 | 0.9996 | 0.0525 | 0.9815 | 0.0097 | 0.9859 |
| P48 | 0.0099 | 0.9575 | 0.0023 | 0.9403 | 0.0455 | 0.9636 | 0.0352 | 1.0038 | -0.0197 | 0.9224 | -0.0202 | 0.9788 |
| P49 | 0.0052 | 0.9830 | 0.0186 | 0.9756 | -0.0016 | 0.9300 | 0.0082 | 1.0060 | -0.0041 | 0.9374 | 0.0060 | 0.9935 |
| P50 | -0.0484 | 1.0233 | 0.0715 | 1.0835 | -0.0383 | 0.9672 | -0.0604 | 1.0587 | -0.0551 | 0.9916 | -0.0062 | 0.9724 |
| P51 | -0.0133 | 0.9896 | 0.0222 | 0.9807 | -0.0172 | 0.9449 | 0.0055 | 1.0069 | -0.0367 | 0.9733 | -0.0039 | 0.9949 |
| P52 | 0.0290 | 0.9749 | -0.0219 | 0.9577 | 0.0027 | 0.9030 | 0.0175 | 0.9838 | 0.0250 | 0.9213 | -0.0095 | 0.9686 |
| P54 | -0.0237 | 0.9872 | 0.0068 | 0.9565 | -0.0323 | 0.9608 | 0.0086 | 0.9949 | -0.0616 | 1.0177 | -0.0289 | 1.0068 |
| P55 | 0.0138 | 0.9492 | -0.0100 | 0.9348 | -0.0070 | 0.8969 | -0.0198 | 0.9763 | -0.0073 | 0.9030 | -0.0248 | 0.9748 |
| P56 | 0.0076 | 0.9540 | -0.0479 | 1.0107 | -0.0390 | 0.9613 | 0.0120 | 0.9787 | 0.0025 | 0.9077 | -0.0250 | 0.9826 |
| P58 | -0.0165 | 0.9632 | -0.0227 | 0.9579 | -0.0375 | 0.9590 | 0.0230 | 0.9883 | -0.0609 | 1.0013 | -0.0387 | 1.0103 |
| P59 | -0.0005 | 0.9567 | -0.0163 | 0.9516 | -0.0310 | 0.9444 | -0.0132 | 0.9832 | -0.0376 | 0.9494 | -0.0225 | 0.9826 |
| P60 | 0.0135 | 0.9874 | 0.0142 | 0.9729 | 0.0051 | 0.9302 | 0.0025 | 1.0064 | 0.0096 | 0.9381 | 0.0030 | 0.9941 |
| P61 | 0.0391 | 1.0026 | 0.0031 | 0.9519 | 0.0141 | 0.9181 | -0.0100 | 0.9914 | 0.0516 | 0.9741 | 0.0222 | 0.9901 |
| P62 | 0.0939 | 1.1581 | -0.0184 | 0.9556 | 0.0762 | 1.0749 | 0.0277 | 1.0058 | 0.1192 | 1.1864 | 0.0740 | 1.1143 |
| P63 | 0.0079 | 0.9878 | 0.0217 | 0.9827 | 0.0132 | 0.9364 | 0.0250 | 1.0215 | 0.0042 | 0.9401 | 0.0163 | 0.9978 |
| P64 | 0.0287 | 0.9581 | -0.0272 | 0.9496 | -0.0241 | 0.9133 | -0.0109 | 0.9638 | 0.0131 | 0.8965 | -0.0036 | 0.9490 |
| P65 | 0.0580 | 0.9684 | 0.0170 | 0.8761 | 0.0407 | 0.8872 | 0.0483 | 0.9540 | 0.1203 | 1.1382 | 0.0999 | 1.1644 |
| P66 | 0.0296 | 0.9762 | -0.0466 | 1.0096 | -0.0193 | 0.9212 | 0.0276 | 0.9944 | 0.0309 | 0.9284 | -0.0042 | 0.9664 |
| P67 | -0.0340 | 0.9820 | 0.0810 | 1.1192 | -0.0401 | 0.9619 | -0.0239 | 0.9827 | -0.0617 | 0.9993 | 0.0122 | 0.9630 |
| P68 | 0.0590 | 1.0233 | 0.0313 | 0.9907 | 0.1425 | 1.4184 | 0.0505 | 1.0142 | 0.0515 | 0.9496 | 0.0314 | 0.9771 |
| P70 | 0.0920 | 1.1475 | 0.0161 | 0.9450 | 0.1242 | 1.3170 | 0.0664 | 1.0655 | 0.1113 | 1.1513 | 0.0598 | 1.0618 |

**Table S10**

Correlations and grade of MIV values between common peaks and the antioxidant, anti-NSCLC activities, respectively

| Peaks | DPPH |  | FRAP |  | CCK-8 |  | Woung healing |  | Transwell migration |  | Transwell invasion |  |
| --- | --- | --- | --- | --- | --- | --- | --- | --- | --- | --- | --- | --- |
|  | MIV | Rank | MIV | Rank | MIV | Rank | MIV | Rank | MIV | Rank | MIV | Rank |
| P1 | 0.0674 | 1 | 0.1141 | 1 | 0.0190 | 20 | -0.2780 | 56 | 0.0563 | 6 | 0.1338 | 1 |
| P2 | 0.0121 | 6 | -0.0059 | 34 | -0.0165 | 36 | 0.0055 | 35 | -0.0277 | 49 | -0.0641 | 56 |
| P4 | 0.0071 | 15 | 0.0492 | 2 | 0.0851 | 4 | -0.4398 | 57 | -0.1069 | 58 | -0.0482 | 52 |
| P5 | 0.0117 | 7 | -0.0181 | 44 | -0.0057 | 29 | -0.1358 | 52 | 0.0864 | 1 | 0.0829 | 7 |
| P6 | -0.0024 | 37 | -0.0034 | 30 | 0.0151 | 22 | -0.0473 | 46 | 0.0050 | 24 | 0.0228 | 20 |
| P7 | 0.0057 | 16 | 0.0018 | 23 | -0.0360 | 43 | 0.1924 | 9 | 0.0259 | 14 | 0.0426 | 15 |
| P8 | 0.0243 | 3 | -0.0182 | 45 | 0.0815 | 5 | 0.0519 | 23 | 0.0015 | 26 | 0.0211 | 21 |
| P9 | -0.0020 | 36 | -0.0210 | 48 | -0.0438 | 47 | 0.0023 | 36 | -0.0138 | 33 | 0.0527 | 11 |
| P10 | -0.0028 | 40 | 0.0040 | 21 | 0.0487 | 10 | -0.0206 | 42 | -0.0172 | 39 | 0.0480 | 13 |
| P12 | -0.0062 | 53 | -0.0080 | 36 | -0.0245 | 39 | -0.0674 | 50 | -0.0159 | 36 | -0.0003 | 35 |
| P13 | 0.0093 | 13 | -0.0273 | 55 | 0.0005 | 27 | 0.0633 | 19 | 0.0045 | 25 | 0.0356 | 16 |
| P14 | 0.0032 | 24 | 0.0140 | 11 | -0.0368 | 44 | 0.1451 | 12 | 0.0138 | 21 | 0.0159 | 25 |
| P15 | 0.0005 | 29 | -0.0106 | 39 | -0.0082 | 30 | -0.0478 | 47 | -0.0056 | 30 | 0.0161 | 23 |
| P16 | -0.0031 | 42 | -0.0149 | 42 | 0.0307 | 16 | -0.0137 | 41 | -0.0065 | 32 | 0.0048 | 29 |
| P17 | 0.0032 | 25 | 0.0097 | 15 | 0.0695 | 6 | 0.0242 | 28 | 0.0640 | 5 | -0.0446 | 50 |
| P18 | -0.0025 | 38 | -0.0104 | 38 | -0.0146 | 35 | 0.0615 | 20 | -0.0195 | 43 | -0.0266 | 43 |
| P19 | -0.0061 | 51 | 0.0050 | 20 | 0.0052 | 24 | 0.0240 | 29 | -0.0230 | 46 | 0.0250 | 19 |
| P20 | -0.0052 | 47 | -0.0215 | 49 | -0.0717 | 56 | 0.0491 | 24 | -0.0168 | 38 | -0.0059 | 37 |
| P21 | -0.0017 | 33 | 0.0165 | 9 | -0.0244 | 38 | 0.1256 | 14 | 0.0257 | 16 | 0.0869 | 6 |
| P22 | -0.0062 | 52 | -0.0008 | 27 | 0.0382 | 13 | -0.0103 | 40 | 0.0662 | 4 | -0.0183 | 41 |
| P23 | 0.0023 | 28 | -0.0075 | 35 | 0.0616 | 8 | 0.3338 | 1 | -0.0025 | 29 | 0.0657 | 9 |
| P24 | 0.0127 | 5 | -0.0222 | 51 | 0.0925 | 2 | 0.0358 | 26 | 0.0372 | 10 | 0.0451 | 14 |
| P25 | -0.0063 | 54 | 0.0086 | 17 | -0.0483 | 51 | -0.0347 | 44 | 0.0309 | 13 | 0.1168 | 3 |
| P26 | 0.0077 | 14 | -0.0266 | 54 | -0.0799 | 57 | 0.0197 | 32 | 0.0246 | 17 | -0.0392 | 48 |
| P28 | 0.0041 | 19 | -0.0108 | 40 | 0.0108 | 23 | 0.2473 | 3 | 0.0173 | 20 | 0.0116 | 27 |
| P29 | -0.0032 | 43 | 0.0135 | 12 | -0.0447 | 48 | -0.2154 | 55 | 0.0008 | 27 | -0.0389 | 47 |
| P30 | 0.0039 | 22 | 0.0053 | 19 | 0.0231 | 18 | 0.1160 | 15 | -0.0672 | 55 | 0.0197 | 22 |
| P32 | 0.0056 | 17 | -0.0048 | 32 | -0.0127 | 34 | 0.0224 | 30 | -0.0162 | 37 | -0.0130 | 38 |
| P34 | 0.0002 | 30 | -0.0529 | 59 | 0.1590 | 1 | 0.1593 | 11 | -0.0146 | 34 | 0.1309 | 2 |
| P35 | 0.0240 | 4 | -0.0368 | 57 | -0.1454 | 59 | 0.2271 | 4 | -0.0534 | 53 | 0.0752 | 8 |
| P36 | -0.0053 | 48 | -0.0025 | 29 | -0.0038 | 28 | 0.0001 | 37 | -0.0390 | 51 | 0.0337 | 17 |
| P37 | -0.0061 | 50 | 0.0092 | 16 | 0.0014 | 26 | -0.0607 | 48 | 0.0477 | 7 | -0.0159 | 39 |
| P38 | -0.0026 | 39 | -0.0049 | 33 | -0.0459 | 49 | 0.0614 | 22 | -0.0443 | 52 | 0.0027 | 32 |
| P39 | -0.0029 | 41 | 0.0169 | 8 | -0.0421 | 46 | -0.0013 | 38 | -0.0598 | 54 | 0.0321 | 18 |
| P42 | 0.0116 | 8 | 0.0074 | 18 | 0.0359 | 15 | 0.2092 | 6 | -0.0183 | 42 | 0.0048 | 30 |
| P43 | 0.0108 | 10 | 0.0211 | 7 | -0.0104 | 33 | 0.2137 | 5 | -0.0155 | 35 | 0.0132 | 26 |
| P44 | -0.0058 | 49 | 0.0235 | 6 | 0.0251 | 17 | 0.0060 | 34 | 0.0348 | 12 | -0.1044 | 59 |
| P46 | 0.0114 | 9 | 0.0007 | 25 | 0.0373 | 14 | -0.0307 | 43 | -0.0063 | 31 | 0.0067 | 28 |
| P47 | 0.0046 | 18 | -0.0190 | 46 | -0.0349 | 42 | -0.1366 | 53 | -0.0008 | 28 | -0.0519 | 54 |
| P48 | -0.0046 | 45 | -0.0233 | 53 | 0.0662 | 7 | 0.0992 | 16 | 0.0079 | 23 | -0.0045 | 36 |
| P49 | 0.0034 | 23 | -0.0410 | 58 | 0.0427 | 12 | 0.0615 | 21 | 0.0371 | 11 | 0.0903 | 4 |
| P50 | 0.0101 | 12 | 0.0131 | 13 | -0.0602 | 53 | -0.0076 | 39 | -0.0705 | 56 | -0.0367 | 46 |
| P51 | -0.0207 | 59 | 0.0002 | 26 | -0.0300 | 41 | 0.1614 | 10 | -0.0196 | 44 | 0.0009 | 34 |
| P52 | -0.0090 | 57 | 0.0143 | 10 | -0.0808 | 58 | -0.5690 | 58 | 0.0130 | 22 | 0.0872 | 5 |
| P54 | -0.0042 | 44 | 0.0277 | 4 | 0.0852 | 3 | 0.1284 | 13 | -0.0241 | 47 | -0.0331 | 45 |
| P55 | -0.0071 | 56 | -0.0227 | 52 | 0.0435 | 11 | -0.0461 | 45 | -0.0181 | 41 | -0.0293 | 44 |
| P56 | 0.0041 | 21 | -0.0015 | 28 | 0.0534 | 9 | 0.0246 | 27 | -0.0213 | 45 | 0.0161 | 24 |
| P58 | -0.0065 | 55 | -0.0103 | 37 | 0.0231 | 19 | 0.0204 | 31 | -0.0276 | 48 | -0.0163 | 40 |
| P59 | 0.0024 | 27 | -0.0190 | 47 | -0.0288 | 40 | -0.7862 | 59 | -0.0282 | 50 | 0.0016 | 33 |
| P60 | -0.0144 | 58 | -0.0288 | 56 | -0.0618 | 54 | 0.1996 | 7 | 0.0667 | 3 | -0.0643 | 57 |
| P61 | -0.0011 | 31 | -0.0117 | 41 | 0.0159 | 21 | 0.0370 | 25 | 0.0423 | 8 | 0.0518 | 12 |
| P62 | 0.0041 | 20 | -0.0215 | 50 | 0.0049 | 25 | 0.0170 | 33 | 0.0259 | 15 | 0.0548 | 10 |
| P63 | -0.0052 | 46 | 0.0353 | 3 | -0.0480 | 50 | -0.1641 | 54 | 0.0380 | 9 | -0.0510 | 53 |
| P64 | 0.0027 | 26 | 0.0011 | 24 | -0.0166 | 37 | 0.1949 | 8 | 0.0234 | 18 | -0.0455 | 51 |
| P65 | -0.0018 | 34 | -0.0044 | 31 | -0.0416 | 45 | -0.0644 | 49 | 0.0193 | 19 | -0.0430 | 49 |
| P66 | -0.0016 | 32 | 0.0240 | 5 | -0.0519 | 52 | -0.0878 | 51 | -0.0172 | 40 | -0.0594 | 55 |
| P67 | -0.0018 | 35 | 0.0122 | 14 | -0.0086 | 31 | 0.0855 | 17 | -0.1036 | 57 | -0.0221 | 42 |
| P68 | 0.0104 | 11 | 0.0031 | 22 | -0.0087 | 32 | 0.3278 | 2 | -0.1584 | 59 | -0.0856 | 58 |
| P70 | 0.0303 | 2 | -0.0176 | 43 | -0.0667 | 55 | 0.0786 | 18 | 0.0765 | 2 | 0.0028 | 31 |
